# Biobeam—Multiplexed wave-optical simulations of light-sheet microscopy

**DOI:** 10.1371/journal.pcbi.1006079

**Published:** 2018-04-13

**Authors:** Martin Weigert, Kaushikaram Subramanian, Sebastian T. Bundschuh, Eugene W. Myers, Moritz Kreysing

**Affiliations:** 1 Max Planck Institute of Molecular Cell Biology and Genetics, Dresden, Germany; 2 Center for Systems Biology Dresden, Dresden, Germany; 3 Faculty of Computer Science, Technische Universität Dresden, Germany; Oxford University, UNITED KINGDOM

## Abstract

Sample-induced image-degradation remains an intricate wave-optical problem in light-sheet microscopy. Here we present *biobeam*, an open-source software package that enables simulation of operational light-sheet microscopes by combining data from 10^5^–10^6^ multiplexed and GPU-accelerated point-spread-function calculations. The wave-optical nature of these simulations leads to the faithful reproduction of spatially varying aberrations, diffraction artifacts, geometric image distortions, adaptive optics, and emergent wave-optical phenomena, and renders image-formation in light-sheet microscopy computationally tractable.

## Author summary

Modern microscopes permit to acquire high quality images of large fields of view, which is the result of a decade-long development of computer aided optical design. However, this high image quality can only be obtained at the very surface of biological specimens: when trying to penetrate deeper into biological tissues, light scattering by cells rapidly leads to severe image blur and computers have so far been unable to model the process by which light forms images in such turbid optical environments. We developed a software that allows one to simulate how microscopes record images deep inside scattering biological samples. Our software reproduces a wide range of optical effects that underlie image blur in tissues. Hence strategies to improve image quality within three-dimensional samples can now be systematically tested by computers. Specifically, our software reproduces intricate wave-optical effects that have recently been proposed as strategies to gain perfect images even in the most turbid environments.This provides the chance for a new generation of microscopes, in which computer models guide the imaging process to enable highest possible resolution even deep inside biological specimens.

This is a *PLOS Computational Biology* Software paper.

## Introduction

Light-sheet fluorescence microscopy is a popular tool for the volumetric imaging of developing organisms [1–5]. As light-sheet microscopes continue to be developed for progressively bigger biological samples [6, 7], there is an increasing need for computers to process gigantic sets of imaging data and extract biologically relevant information [8]. With sample size, however, also light-scattering induced imaging artifacts become increasingly prevalent. During data acquisition as well as post-processing, these imaging artifacts are mostly dealt with on a purely phenomenological basis. A faithful forward model of the wave-optical imaging process, however, would *i)* enable rigorous benchmarks of deconvolution and segmentation strategies against ground-truth data [9], *ii)* serve as training platforms for machine learning approaches for image restoration and information extraction and *iii)* leverage the efficient use of adaptive optics [10, 11] to prevent sample-induced image degradation during the acquisition process. Predicting light-tissue interactions is particularly demanding when leaving the single scattering regime [12] or strictly diffusive transport [13]. And despite significant computational advances [14], generally applicable solutions [15] remain computationally costly, effectively prohibiting the simulation of image formation in microscopy. Even when constraining simulations to the biological relevant case of predominantly forward scattering tissue [10, 16], individual point spread function (PSF) calculations still require multiple seconds [17]. As aberrations deep inside tissues are unique for virtually each point in the sample [10, 12] a realistically large biological specimen, i.e. an embryo with a volume ∼ 100 μm^3^ would require 10^5^–10^6^ volumetric PSF calculations in order to faithfully mimic the wave-optical imaging process, which with current methods would take several weeks. As a consequence, attempts to simulate microscopic imaging have been limited to ray optics [18] or convolution with a constant PSF [19], approaches that do not reflect the wave-optical nature of light interaction with optically heterogeneous biological samples. Here, we report on *biobeam*, a software package that enables the first rigorous simulations of wave-optical image formation in light-sheet microscopes by *i)* a novel multiplexing scheme for PSF calculations and *ii)* efficient GPU parallelization.

## Design and implementation

The pipeline underlying our software is based on the observation that the beam-propagation model (BPM) for fiber optics [20, 21] can also be used to mimic scattering biological cells [22]. To guarantee good accuracy of BPM beyond strictly paraxial wave propagation, we use the exact propagator together with a locally adapted expansion of refractive indices (see S1 Text, Notes 1 and 4 for details and validation against analytically tractable scattering models). To massively reduce the computational cost for ∼10^6^ wave-optical PSF calculations, we introduce a novel multiplexing scheme. In this we exploit the fact that for typical imaging scenarios, a single camera image can be constructed from sets of 100s to 1000s of mutually independent, spatially varying, non-overlapping PSFs. Such sets can be calculated within single, highly multiplexed simulations (see S3 Fig and S1 Text, Note 8), which is similar to the operational principle of highly multiplexed confocal measurements of spinning disc microscopes [23, 24]. Our software is further accelerated ∼20 fold by the efficient use of GPU implementations for all low-level calculations. In this way, the propagation of an arbitrary light field such as the multiplexed set of ∼1000 PSFs through a typical volume of 1024^3^ voxels takes less than 500ms on a single graphics card (cf. S1 Text, Note 5, S1 Table and S4 Video), corresponding to a 20.000 fold acceleration compared to the sequential calculation of PSFs. Implementation was done as an open source Python software package. All computationally heavy parts are lifted to the GPU via OpenCL [25], thereby keeping all the advantages of Python as a dynamically typed high level language that is vastly used in the scientific community without compromising on performance. Apart from its technical focus on speed, *biobeam* is specifically designed to make wave-optical experiments in-silico as easy as possible, e.g. by providing a simple API that makes it easy to apply different input fields as well as PSF/aberration calculations via diffraction-limited point source propagation through tissue (see S1 Text, Note 3 for example listings).

## Results

### Light-sheet microscopy

**Fig 1:**
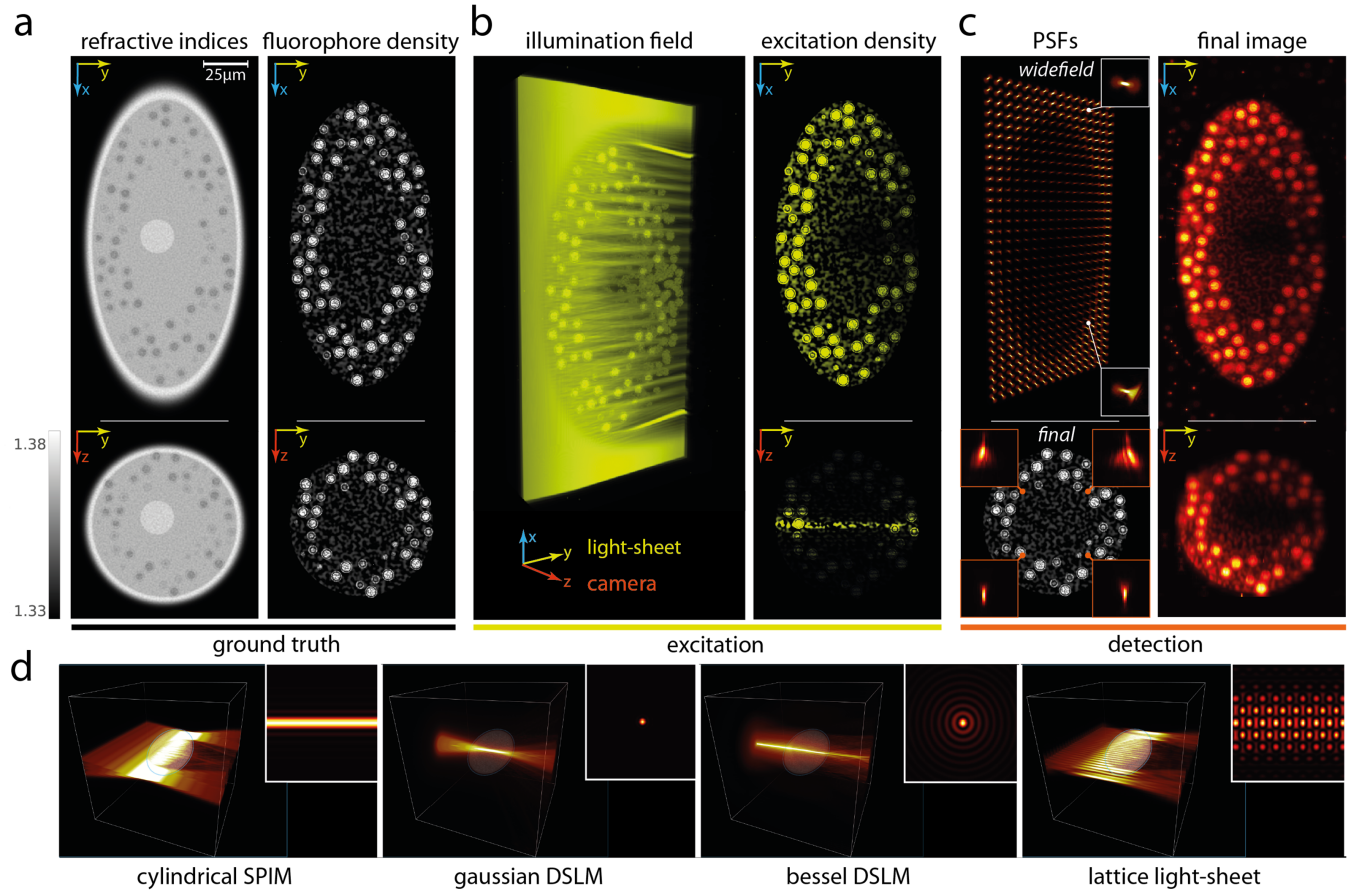
Rigorous wave-optical simulation of image formation process in light-sheet microscopy. (a) Synthetic tissue phantom of a multicellular organism (100 × 200 × 100*μm*) comprising a complex refractive index distribution (left, *n* = 1.33–1.38) and a fluorophore distribution of interest (right). (b) Wave optical simulation of the illuminating light sheet and resulting excitation distribution within the sample at a given z position. (c) Partially coherent simulation of the detection path by multiplexed calculation of all independent point spread functions (left) and the resulting simulated camera image combining illumination and fluorescence path of light through the scattering sample. (d) Alternative light-sheet modalities (see also S1 and S5 Videos and main text for details).

**Fig 2:**
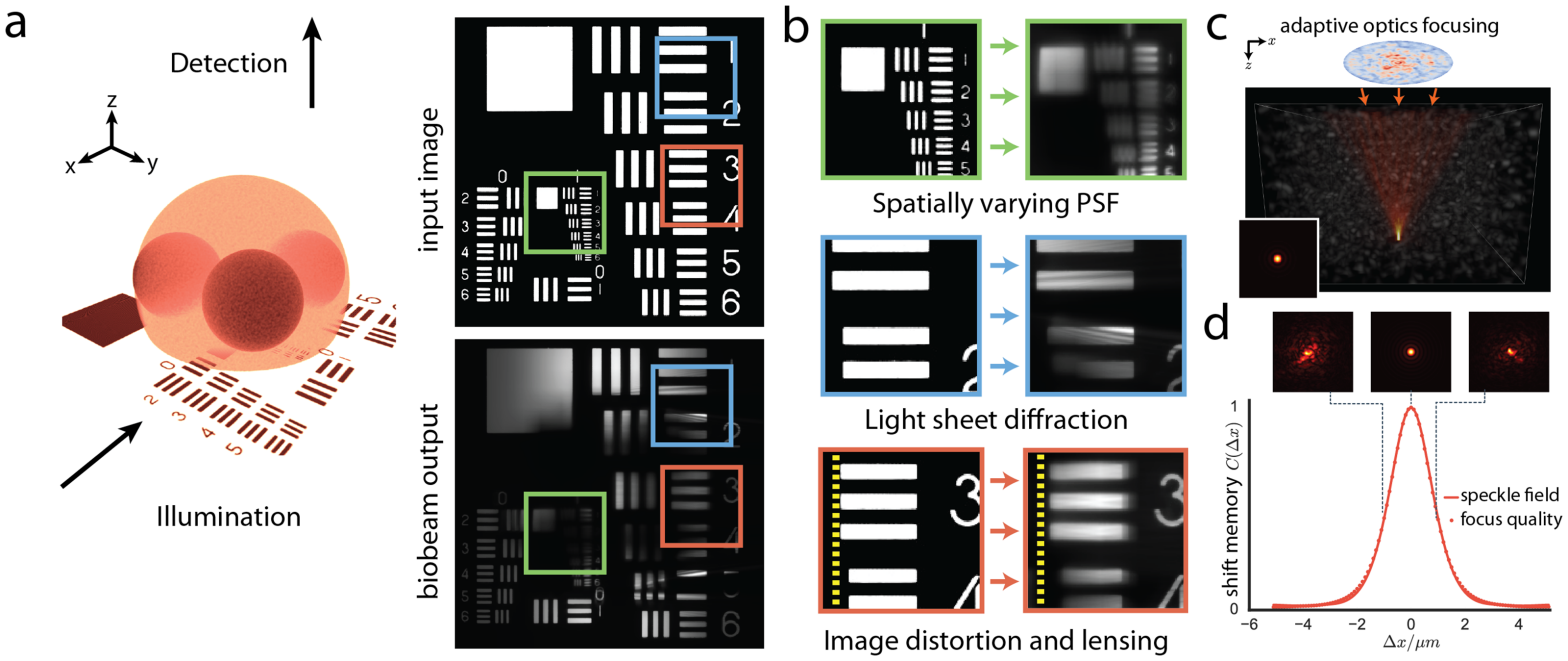
Optical capabilities of the biobeam image-formation pipeline. (a) A test chart at the mid-section of an optically heterogeneous embryo-model (*n* = 1.35–1.39 diameter 140*μm*) is illuminated by cylindrical light sheet (*NA* = 0.15), and imaged from an orthogonal position (*NA* = 0.6). (b) Details of these wave-optically calculated images reveal *i)* spatially varying image blur, contrast loss and absorption induced by the heterogeneity of the sample, *ii)* diffraction artifacts from the light-sheet-typical coherent illumination, and *iii)* geometric image distortions such as lensing, split-screen type image distortions, and object displacements lensing. (c) *Biobeam* is further capable of adaptive optics simulations by which reversal of guide star emitted light fields yields perfect foci in scattering tissues. (d) Adaptive optics simulations faithfully reproduces the shift-shift memory effect, an emergent wave-optical phenomenon, here at 4 mean-free-paths deep inside the tissue.

We first demonstrate the power of our software package by presenting the first wave-optical simulation of the volumetric image-formation process in a light-sheet microscope [5, 9, 18]. To start, a cylindrical sheet of light is propagated at a specific axial position through a fluorescently labelled embryo model with refractive indices of cytoplasm, nuclei and organelles as previously reported [12] (Fig 1a and S1 & S7 Videos). From this, one obtains the fluorescence excitation at every point in the volume for every axial position of the incident light field. Next, for each focal plane a full set of detection PSFs is obtained by propagating light from multiplexed, diffraction-limited point-sources orthogonally through an idealized, refocusing lens towards the camera (Fig 1c, upper left, S2 Video). This way, we obtain a quasi-continuum of spatially-dependent, volumetric PSFs with position-dependent aberrations that stem from distortions and scattering in both the illumination and the detection paths (Fig 1c left, see also S4 Fig for calculated aberration maps). Convolving the exhaustively sampled sets of spatially-varying PSFs with the fluorescent object finally yields the wave-optical image as seen by the camera (Fig 1c, right). These simulations are particularly demanding due to the large grid size (i.e. 1024 × 2048 × 1024 voxels for Fig 1) and sampling density requirements (typically 0.5–5*μm*), here resulting in a total of 10^6^ PSFs that were calculated in well under 10 minutes on a single graphics card (see also S1 and S2 Videos). In contrast, the non-multiplexed calculations would have required more than two months on a single CPU. As a result of this computational pipeline, *biobeam* generates faithfully calculated 3D microscopy data sets that account for both refraction and diffraction based imaging artifacts. These include image blur and contrast loss, spatially varying PSFs, speckle artifacts, image granularity, as well as sample-induced geometric distortions such as lensing, image displacements and split-screen type double images (cf. Fig 2a and 2b, S3 Video and S1 Fig for benchmarks against analytically tractable models).

We additionally validated our approach by comparing simulation results for several experimental situations, such as the diffraction pattern behind an edge knife (S5 Fig), the scattering of a light-sheet at agarose embedded micro-spheres (S6 Fig) and the image distortions obtained when projecting test images through a refracting sphere (S7 and S8 Figs). Beyond the theoretical validation of the core propagation model, these successful comparisons of simulations against experiments further demonstrate that our high-level implementation works correctly in the biological relevant case of low refractive index contrasts.

Being a particularly flexible software package, *biobeam* provides further pre-implemented illumination modalities, including Bessel lattices (Fig 1d, right, see S2 Fig, S5 Video), and allows for practical extraction of sample-induced aberrations as spatially resolved Zernike maps (see S4 Fig and S1 Text, Note 9).

### Adaptive optics simulations

Next, we demonstrate *biobeam*’s capability to accurately simulate wave-optical effects relevant to adaptive optics (AO) imaging [10]. It is well-established and exploited in imaging that perfect imaging foci can be created behind strongly scattering screens [26] and within biological samples by appropriately shaping the wavefront before entry into the scattering medium. We recapitulated this finding by explicit simulations of light propagating through a spatially extended synthetic tissue sample represented by a Perlin-noise refractive index distribution (*n* = 1.36 ± 0.03). As expected, we find also in simulations that conjugation of the wavefront at the surface of the sample allows one to generate diffraction-limited foci inside these scattering tissues (Fig 2c, S6 Video and S9 Fig). Furthermore, we show that *biobeam* is capable of reproducing the shift-shift memory effect, an emergent wave-optical phenomenon responsible for the significant robustness of adaptive imaging against lateral focus displacements. According to this wave-optical phenomenon, the radius of the iso-planatic patch (distance over which an AO correction pattern works) is determined by the statistics of a tissue generated speckle field inside this medium. Based on a total of 22500 PSF calculations, we show that our simulations faithfully capture this emergent wave-optical effect. In agreement with recent analytical arguments and their empirical confirmation [10], we show by our computational microscopy experiments that the average persistence of the laterally shifted focus is precisely limited by the autocorrelation length of a speckle pattern that would result from an incident plane wave. This phenomenon is accurately reproduced even four mean-free-path lengths inside the tissue (Fig 2d). While currently using a variant of the beam propagation method, our PSF multiplexing scheme is in principle also compatible with other low-level field stepping algorithms, including those that iteratively account for multiple back-reflections [14, 15], when even higher precision or penetration depths are required.

## Discussion

We summarize that *biobeam* enables faithful whole-tissue wave-optical simulations of light-sheet microscopes due to i) a novel multiplexing scheme of PSF calculations and ii) efficient GPU parallelization. *Biobeam* renders the biological imaging process computationally tractable, thus providing the link between wave-optically recorded image material, and the ground truth object. Given the modular nature of our software package, these simulations are easy to implement and can be flexibly adapted to custom imaging scenarios and microscopes. Beyond the reproduction and identification of commonly occurring imaging artifacts (see Supplement for comparison with experimental data), we demonstrated that *biobeam* is compatible with imaging scenarios in which sample-induced PSFs degradation is overcome by the use of adaptive optics. Furthermore, we showcase the optical capabilities and accuracy of our software by explicitly demonstrating that emergent wave-optical phenomena such as the shift-shift memory effect are quantitatively reproduced deep inside tissues. While we chose a variant of BPM as a low-level field stepping routine, the here presented strategy of multiplexing PSF calculation is more general in nature, and may also be used in combination with other light propagation algorithms, e.g. in scenarios where higher accuracy at large angles and/or more isotropic scattering is of relevance [14].

We conclude that *biobeam* is a flexible and particularly powerful platform to systematically study wave-optical image-formation by microscopes in scattering biological tissues.

## Availability and future directions

Prospectively, we see *biobeam* helping to improve microscope design, enhancing deconvolution and segmentation strategies by providing realistic imaging data-sets along with ground truth data, and paving the way for a new generation of smart, adaptive microscopes that learn to treat the sample as a part of the optical path. Of great help in this will be the rapidly increasing knowledge of stereotypical refractive index distributions in embryos, tissues, cells, subcellular compartments and their constituents as derived from tomographic phase microscopy [12, 27–29] and complemented by electron microscopy morphological data [30]. Beyond the simulation of light-sheet microscopes, our software can also be used to simulate other imaging modalities such as wide-field, laser-scanning confocal, and light-field [31] microscopes, as well as for novel micro-lens concepts [32] and the emerging field of soft photonics [33]. Especially, we emphasize the potential to improve the understanding of physiological image-formation inside the eye [34], taking into account the optics of the lens [35] as well as the retina [36–39]. *Biobeam* is available as open-source (BSD-3 license) python package at https://maweigert.github.io/biobeam. Datasets can be found at https://publications.mpi-cbg.de/6874-data/.

## Supporting information

S1 Text 25 pages description of the numerical method, validation, benchmarks, details of the numerical experiments, and example listings. (PDF)

S1 Video Wave-optical simulation of the image-formation process in light-sheet microscopy. The tissue model represents a multicellular (760 nuclei) organism of size (100*μm*, 200*μm*, 100*μm*) in an aqueous medium with *n* = 1.33. The refractive index distribution is in the range *n* ∈ (1.35, 1.42) comprising reference values for cell nuclei, eggshell and the cytoplasm [12]. Weak absorption is homogenously present, but could also be localized (e.g. a spherical absorbing compartment in the center). The simulations of both the illumination and detection processes were carried out on a computational grid of (1024, 2048, 1024) voxels with a spacing of 100*nm* along each dimension. The illumination field is a cylindrical light sheet with *NA*_*illum*_ = 0.1 focused laterally at the center and the detection system was assumed to have *NA*_*detect*_ = 0.6. For generating the final stack both illumination and detection fields were simulated at 200 different axial positions. The deterioration of both resolution and intensity at regions where photons along either the illumination or detection path had to travel through large inhomogeneities can clearly be seen.
(MP4)

S2 Video Illustration of a single PSF calculation inside the tissue via the propagation of analytically defined diffraction-limited input fields. Due to the linearity of wave-optics, these PSF calculations can be highly multiplexed, as firstly exploited by *biobeam*.
(MP4)

S3 Video A *biobeam* generated video illustrating rigorous wave-optical mimicry of a wide-field microscope. The imaging of a 100 μm^2^ test chart is simulated while a refractive sphere is continuously introduced into the microscope’s optical path. *biobeam* generated the underlying wave-optical simulations in 30 seconds.
(MP4)

S4 Video Screencast of an interactive command line session demonstrating *biobeam*’s capabilities and speed. All calculations happen in real time.
(MP4)

S5 Video Video showing the predefined illumination modes and simulated light sheets being scanned through a biologically plausible tissue model. Both coherent (cylindrical lens SPIM) illumination and partially-incoherent illumination modes (time scanned Gaussian/Bessel beams) are simulated.
(MP4)

S6 Video Showing the simulation of a aberration pre-compensated wavefront focusing deep into tissue and the shift-shift memory effect. (MP4)

S7 Video Example of a plane-by-plane illumination of a tissue model mimicking an embryo. (MP4)

S1 Fig Validation of *biobeam* with analytical solutions. a) Plane wave scattered by three solid spheres (λ = 500*nm*, r = 2-2.5*μm*, refractive index contrast m = 1.05), b) Comparison of analytical solution (Mie calculus) versus *biobeam* simulation. c) Error percentage of near field distribution as a function of single sphere radius r (Δ*n* = 0.05) and refractive index contrast Δ*n* (r = 2.5*μm*). d) Left: Phase function of analytically tractable coated spheres as cell models (m = 1.02/1.04, r = 5*μm*/4*μm*) shows high accuracy up to approximately 0.5 radians. Right: size dependent scattering efficiency of the same sphere architecture and its inverse.
(PDF)

S2 Fig Propagation of different predefined input fields through a tissue model of size (100*μm*, 100*μm*, 100*μm*) and grid dimension (1024^3^). The respective pupil function is shown in the upper row.
(PDF)

S3 Fig Detection aberration and PSF calculation. Propagating a diffraction limited input field through parts of the sample and refocusing by an idealized optical system gives the focus field as seen by the detector. If the refocus spots are separated for different starting points, the propagation of a complete grid can be carried out in a highly multiplexed manner, accelerating the process for typical microscopy simulations by a factor 100–1000.
(PDF)

S4 Fig Calculating the aberrations of the detection PSF for a given z plane within a synthetic tissue model. The model’s physical size is (200*μm*, 100*μm*, 100*μm*) and the dimension of the computational grid is (1024, 512, 512). The detection wavelength is λ = 522*nm*, the numerical aperture is *NA* = 0.5 and the aqueous immersion medium has refractive index *n*_0_ = 1.33. The refractive index distribution of the tissue model mimics an eggshell, cell nuclei and granular random fluctuations within the biological plausible range of *n* ∈ (1.35, 1.43).
(PDF)

S5 Fig Diffraction around a knife edge. a) Experimental setup: Light is focused with an incoherent light source (M470L3 Thorlabs, λ = 470*nm*) such that an almost plane wave (*NA* = 0.001) illuminated the knife edge. The diffracting light was imaged below at different depths from the edge. b) The simulation was done on a computational cell of size (1024 × 256 × 1830) with voxel size Δ*x* = 0.29*μm*. We simulated the diffraction in the case of a single plane wave (coherent, top) and the incoherent superposition of 100 random incident plane waves of different small incident angle (corresponding to *NA* = 0.001, bottom). c) The experimental acquired intensity. Scalebar is 12*μm* in both axial and lateral direction (depicted with axial/lateral aspect ratio of 8, due to space constraints). d) Intensity plot at a given axial position (dashed line) for simulation, experiment and the intensity calculated via Fresnel-integral (Theory).
(PDF)

S6 Fig Experimental validation on a commercial light-sheet microscope. a) Poly-methylmethacrylate (PMMA) micro-particles with a diameter of 20*μm* and refractive index of *n* = 1.495 were embedded in an OptiPrep/agarose block (*n* ≈ 1.43) labelled with Alexa Fluor 488. A stationary illuminating light-sheet with a waist of 1.7*μm* and a lateral extension of ≈100*μm* was generated with a LZ1 (Zeiss) light-sheet microscope, incident on the agarose embedded spheres. Stacks were acquired at a step size of 0.414*μm*. b) Simulation results of the intensity distribution behind the sphere at a plane incident to the sphere center. c) Experimental intensity image. Scalebar is 20*μm* in both cases. Dashed lines indicate regions with specific diffraction patterns that the simulation correctly reconstitutes.
(PDF)

S7 Fig Custom micro-projection setup built in our lab, controlled with custom LabView programs. The setup allows for patterns to be micro projected onto a sample with predefined illumination-source, size, magnification and NA of influx optics. The efflux optics allows for the collection of the light and recording on the camera.
(PDF)

S8 Fig Experimental micro-projection of a test-chart through a glass sphere and comparison with simulation. Experimental setup as in S7 Fig. The negative USAF (R1DS1N, Thorlabs) test-chart was illuminated incoherently (M470L3 Thorlabs) and projected behind a glass sphere (Borosilicate material, *n* = 1.48, 110*μm* diameter, Cospheric LLC, USA). The images were captured using an Andor Zyla 5.5 sCMOS camera, while focusing through the sphere (see S7 Fig). Depicted are images from the experiment (Real) and the simulation. The difference images are calculated wrt. to the undistorted test-chart image, showing that the real sphere-induced image distortions are qualitatively reproduced by the simulation.
(PDF)

S9 Fig Simulation of shift-shift memory effect. a) Guide-star assisted diffraction-limited focusing in scattering tissue model. b) Lateral translation of this aberration compensated beam leads to gradual degradation. c) Quantification of focus degradation via correlation *C*(Δ*x*) vs. distance (dotted line) at different penetration depth (in mean free path). Agreement with the correlation function of a scattered plane wave (solid line) is most pronounced at higher penetration depth. Deviation at low penetration depth (1 mfp) agree with experimental observations [10].
(PDF)

S10 Fig Aberration correction. a) A rectilinear ground truth stripe pattern occurs distorted when imaged through a sphere. b) Fitting a radial distortion map to simulation results of this scenario, allows one to fix the aberrations at the region of interest (green) as seen through the spherical cell phantom.
(PDF)

S1 Table Runtimes of different methods for the simulation of a plane wave propagating through a given refractive index distribution. (PDF)
