# Supplementary material for: Biobeam—Multiplexed wave-optical simulations of light-sheet microscopy
Source: PLoS Comput Biol. 2018 Apr 13;14(4):e1006079. doi: 10.1371/journal.pcbi.1006079 (PMC5898703; doi:10.1371/journal.pcbi.1006079)
Supplement: S1 Text — (PDF) [file pcbi.1006079.s001.pdf]

Supplementary Material

Biobeam – Multiplexed wave-optical simulations of light-sheet microscopy

Martin Weigert, Kaushikaram Subramanian, Sebastian T. Bundschuh,
Eugene W. Myers & Moritz Kreysing

March 23, 2018

Supplementary Videos

Video 1: Wave-optical simulation of the image-formation process in light-sheet microscopy: The tissue model represents a multicellular (760 nuclei) organism of size $(100\mu m, 200\mu m, 100\mu m)$ in an aqueous medium with $n = 1.33$. The refractive index distribution is in the range $n \in (1.35, 1.42)$ comprising reference values for cell nuclei, eggshell and the cytoplasm[1]. Weak absorption is homogenously present, but could also be localized (e.g. a spherical absorbing compartment in the center). The simulations of both the illumination and detection processes were carried out on a computational grid of $(1024, 2048, 1024)$ voxels with a spacing of $100nm$ along each dimension. The illumination field is a cylindrical light sheet with $NA_{illum} = 0.1$ focused laterally at the center and the detection system was assumed to have $NA_{detect} = 0.6$. For generating the final stack both illumination and detection fields were simulated at 200 different axial positions. The deterioration of both resolution and intensity at regions where photons along either the illumination detection path had to travel through large inhomogeneities can clearly be seen.

Video 2: Illustration of a single PSF calculation insight the tissue via the propagation of analytically defined diffraction-limited input fields. Due to the linearity of wave-optics, these PSF calculations can be highly multiplex, as firstly exploited by *biobeam*.

Video 3: A *biobeam* generated video illustrating rigorous wave-optical mimicry of a wide-field microscope. The imaging of a $100\mu m^2$ test chart is simulated while a refractive sphere is continuously introduced into the microscope's optical path. *biobeam* generated the underlying wave-optical simulations in 30 seconds.

Video 4: Screencast of an interactive command line session demonstrating *biobeam*'s capabilities and speed. All calculations happen in real time.

Video 5: Video showing the predefined illumination modes and simulated light sheets being scanned through a biological plausible tissue model. Both coherent (cylindrical lens SPIM) illumination and partially-incoherent illumination modes (time scanned Gaussian/Bessel beams) are simulated.

Video 6: Showing the simulation of a aberration pre-compensated wavefront focusing deep into tissue and the shift-shift memory effect.

Video 7: Example of a plane-by-plane illumination of a tissue model mimicking an embryo.

Supplementary Figures

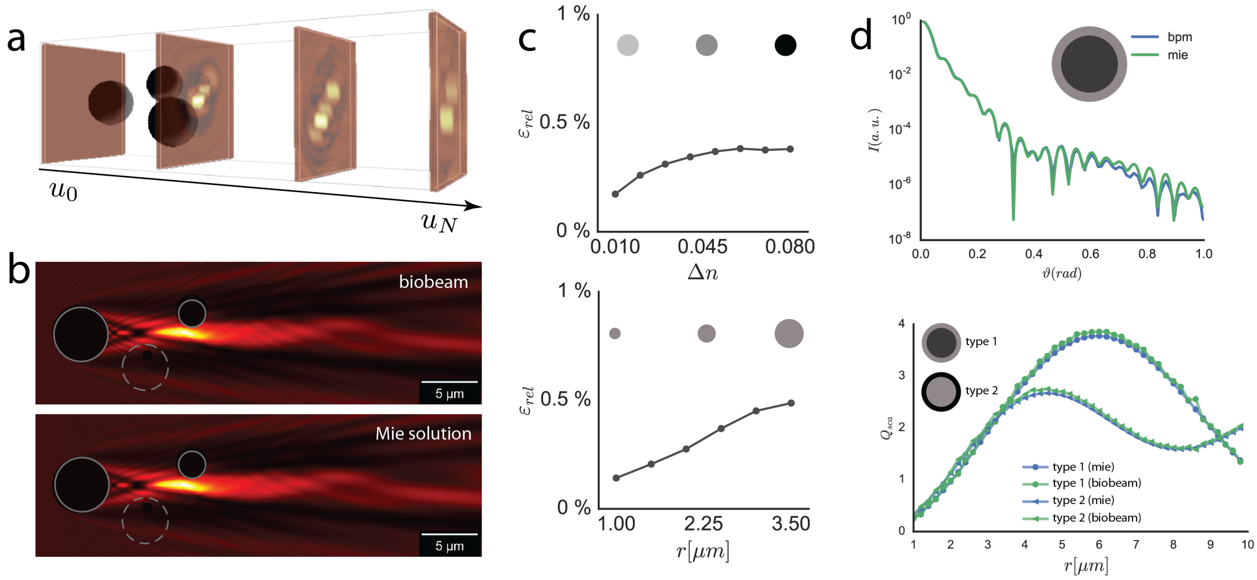

Supplementary Figure 1: Validation of *biobeam* with analytical solutions. a) Plane wave scattered by three solid spheres (λ=500nm, r=2-2.5μm, refractive index contrast m=1.05), b) Comparison of analytical solution (Mie calculus) versus *biobeam* simulation. c) Error percentage of near field distribution as a function of single sphere radius r (Δn = 0.05) and refractive index contrast Δn (r=2.5μm). d) Top: Phase function of analytically tractable coated spheres as cell models (m=1.02/1.04, r=5μm/4μm) shows high accuracy up to approximately 0.5 radians. Bottom: size dependent scattering efficiency of the same sphere architecture and its inverse.

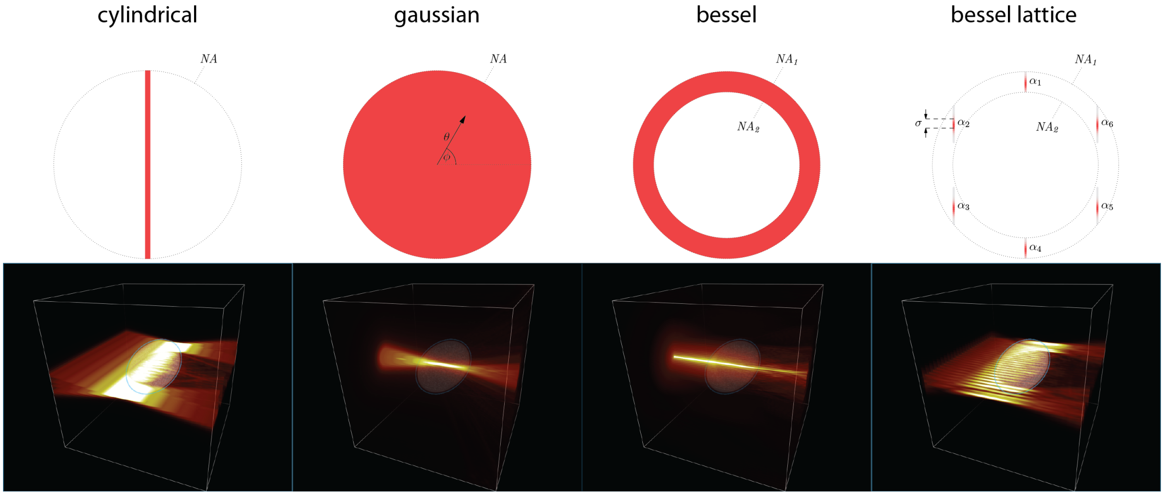

Supplementary Figure 2: Propagation of different predefined input fields through a tissue model of size $(100\mu m, 100\mu m, 100\mu m)$ and grid dimension (1024^3). The respective pupil function is shown in the upper row.

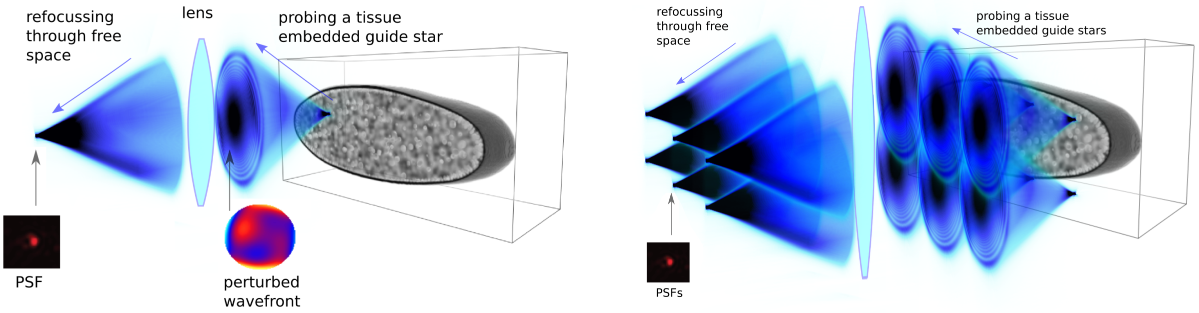

Supplementary Figure 3: Detection aberration and PSF calculation. Propagating a diffraction limited input field through parts of the sample and refocusing by an idealized optical system gives the focus field as seen by the detector. If the refocus spots are separated for different starting points, the propagation of a complete grid can be carried out in a highly multiplexed manner, accelerating the process for typical microscopy simulations by a factor 100–1000.

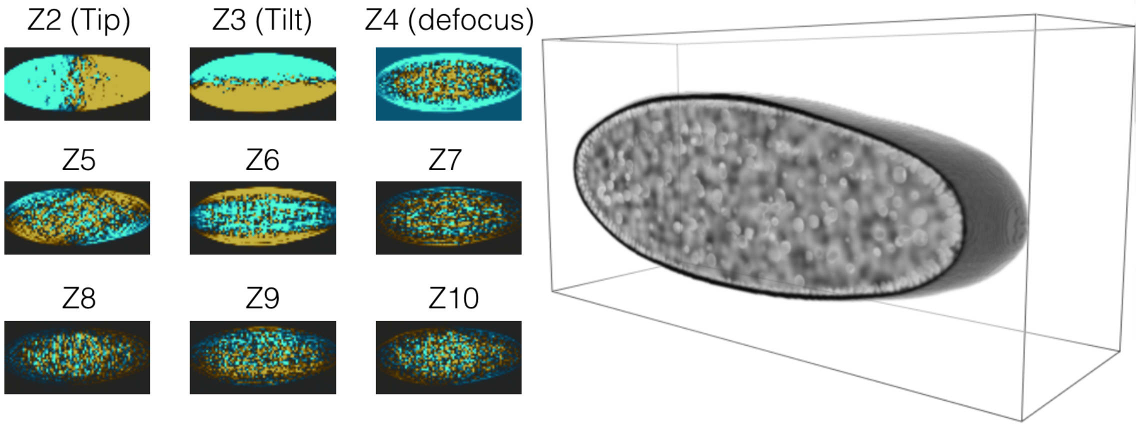

Supplementary Figure 4: Calculating of aberrations of the detection point spread function (PSF) for a given z plane within a synthetic tissue model. The model's physical size is ($200\mu m$, $100\mu m$, $100\mu m$) and the dimensions of the computational grid are $(1024, 512, 512)$. The detection wavelength is $\lambda = 522nm$, the numerical aperture is $NA = 0.5$ and the aqueous immersion medium has a refractive index of $n_0 = 1.33$. The refractive index distribution of the tissue model mimics an eggshell, cell nuclei and granular random fluctuations within the biological plausible range of $n \in (1.35, 1.43)$.

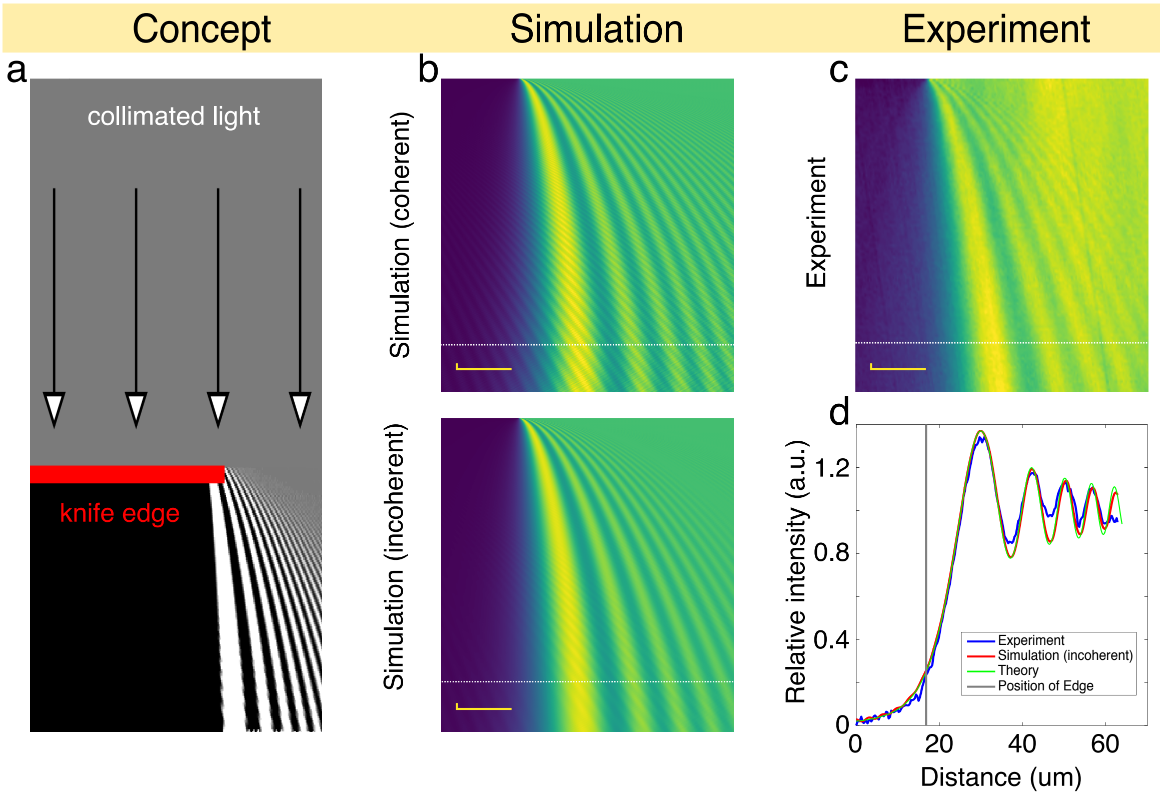

Supplementary Figure 5: Diffraction around a knife edge. a) Experimental setup: Light is focused with an incoherent light source (M470L3 Thorlabs, $\lambda_0 = 470nm$) such that an almost plane wave ($NA = 0.001$) illuminated the knife edge. The diffracting light was imaged below at different depths from the edge. b) The simulation was done on a computational cell of size $(1024 \times 256 \times 1830)$ with voxel size $\Delta x = 0.29\mu m$. We simulated the diffraction in the case of a single plane wave (coherent, top) and the incoherent superposition of 100 incident plane waves of uniformly sampled wavelengths $\lambda \in [460nm, 480nm]$, corresponding to the measured spectral width of $\pm 10nm$ of the light source (incoherent, bottom). c) The experimentally acquired intensity. Scale bar is $12\mu m$ in both axial and lateral direction (depicted with axial/lateral aspect ratio of 8, due to space constraints). d) Intensity plot at a given axial position (dashed line) for simulation, experiment and the intensity calculated via Fresnel-integral (Theory).

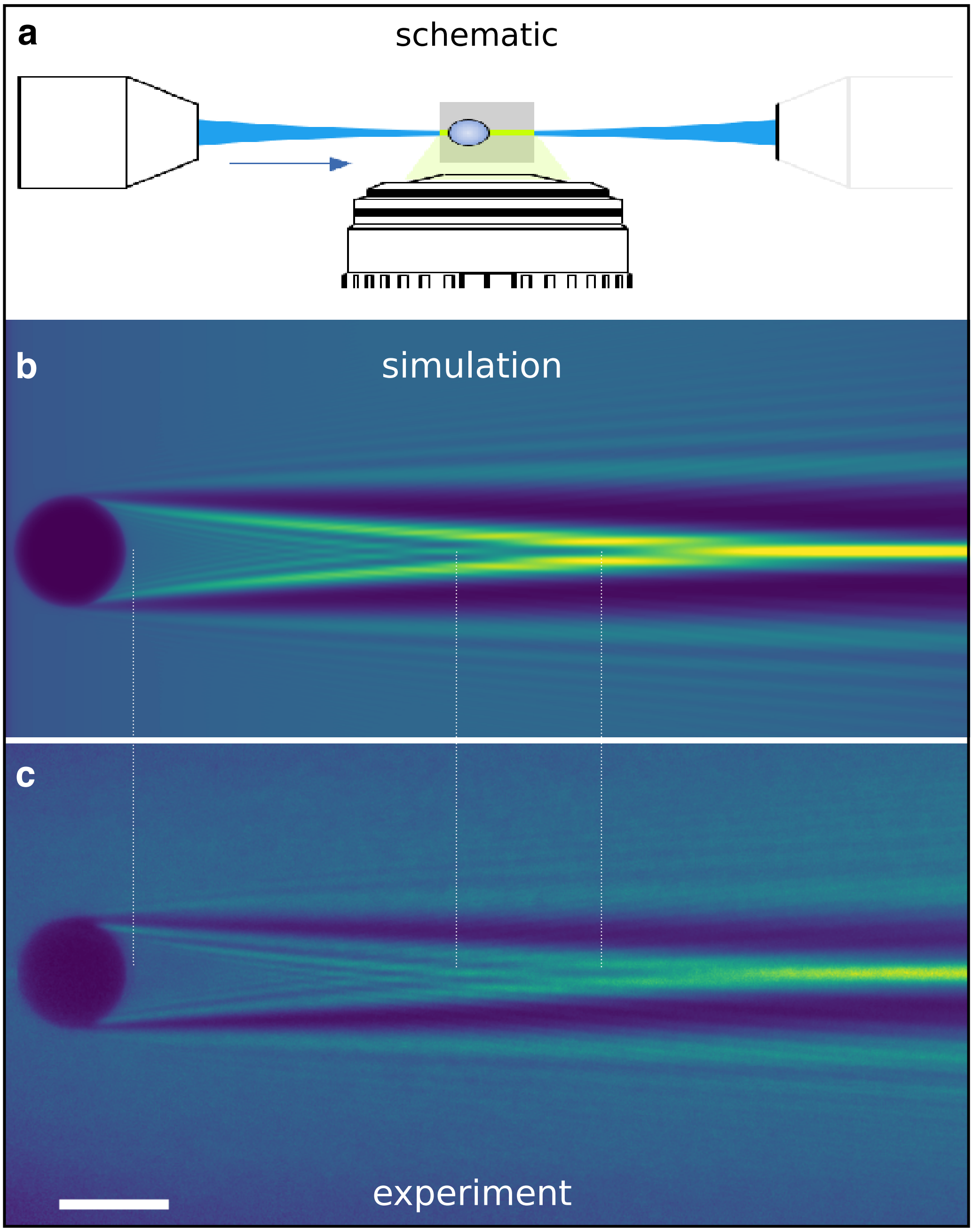

Supplementary Figure 6: Experimental validation on a commercial light-sheet microscope. a) Polymethylmethacrylate (PMMA) microparticles with a diameter of $20\mu m$ and refractive index of $n = 1.495$ were embedded in an block of OptiPrep (Progen Biotechnik GmbH) / agarose (Sigma Aldrich) with refractive index of $n \approx 1.43$) and which was labelled with Alexa Fluor 488. A stationary illuminating light sheet with a waist of $1.7\mu m$ and a lateral extension of $\approx 100\mu m$ was generated with a LZ1 (Zeiss) light-sheet microscope, incident on the agarose embedded sphere. Stacks were acquired at a step size of $0.414\mu m$. b) Simulation results of the intensity distribution behind the sphere at a plane incident to the sphere center. c) Experimental intensity image. Scale bar is $20\mu m$ in both cases. Dashed lines indicate regions with specific diffraction patterns that the simulation correctly reconstitutes.

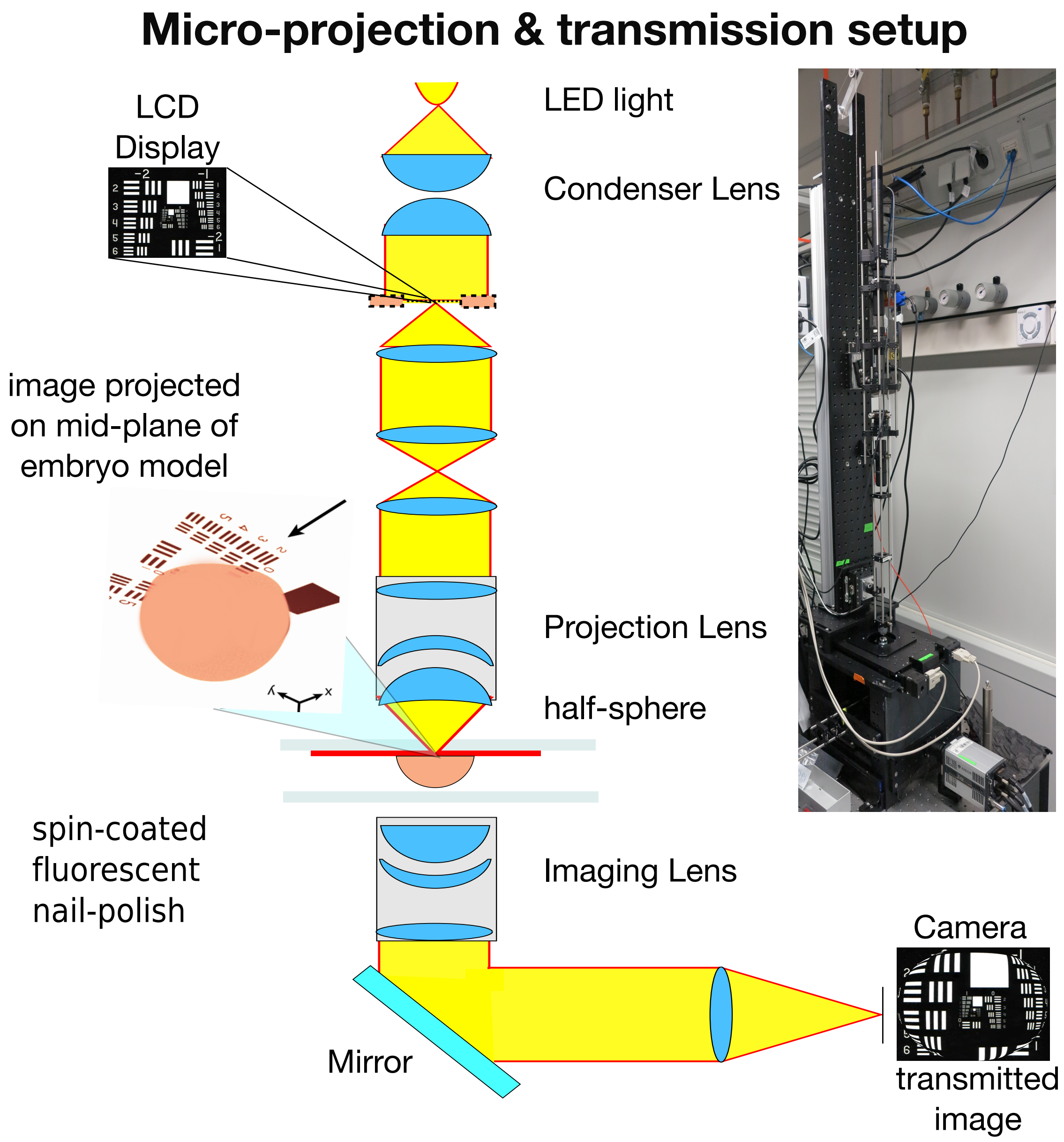

Supplementary Figure 7: Custom micro-projection setup built in our lab, controlled with custom LabView programs. The setup allows for patterns to be micro-projected onto a sample with predefined illumination-source, size, magnification and NA of influx optics. The efflux optics allow for the collection of the light and recording on the camera.

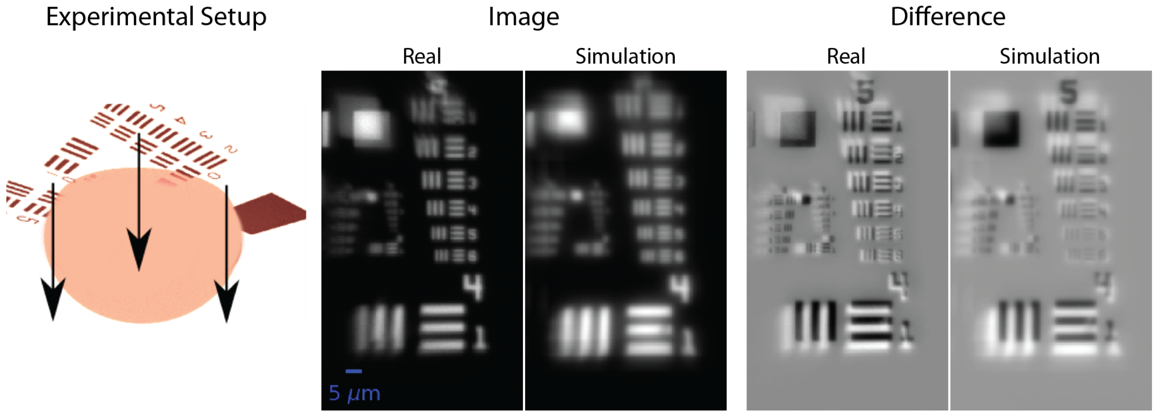

Supplementary Figure 8: Experimental micro-projection of a test-chart through a glass sphere and comparison with simulation. Experimental setup as in Supp. Fig. 7. The negative USAF (R1DS1N, Thorlabs) test-chart was illuminated incoherently (M470L3 Thorlabs) and projected behind a glass sphere (Borosilicate material, $n = 1.48$, $110\mu m$ diameter, Cospheric LLC, USA). The images were captured using an Andor Zyla 5.5 sCMOS camera, while focusing through the sphere (see Supp. Fig. 7). Depicted are images from the experiment (Real) and the simulation. The difference images are calculated w.r.t. to the undistorted test-chart image, showing that the real sphere-induced image distortions are qualitatively reproduced by the simulation.

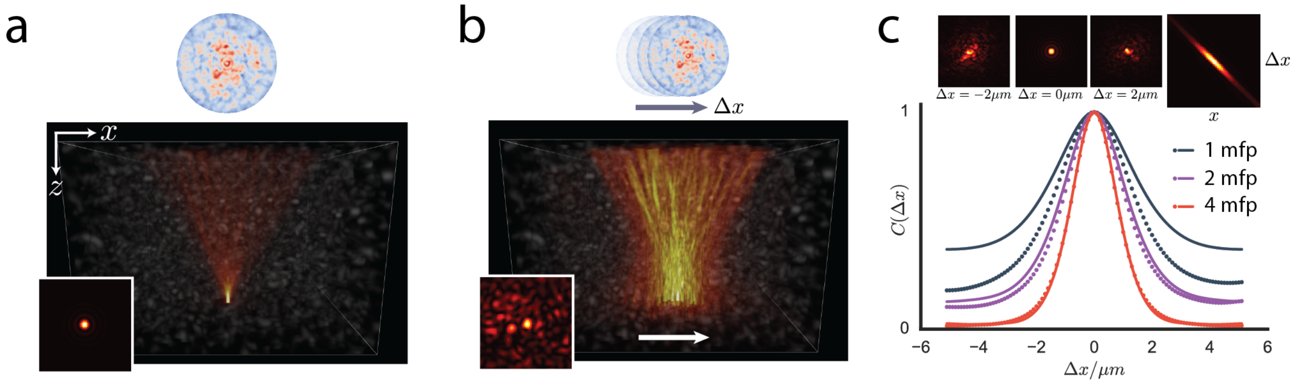

Supplementary Figure 9: Simulation of shift-shift memory effect. a) Guide-star assisted diffraction-limited focusing in scattering tissue model. b) Lateral translation of this aberration compensated beam leads to gradual degradation. c) Quantification of focus degradation via correlation $C(\Delta x)$ vs. distance (dotted line) at different penetration depth (in mean free path). Agreement with the correlation function of a scattered plane wave (solid line) is most pronounced at higher penetration depth. Deviation at low penetration depth (1 mfp) agree with experimental observations [2].

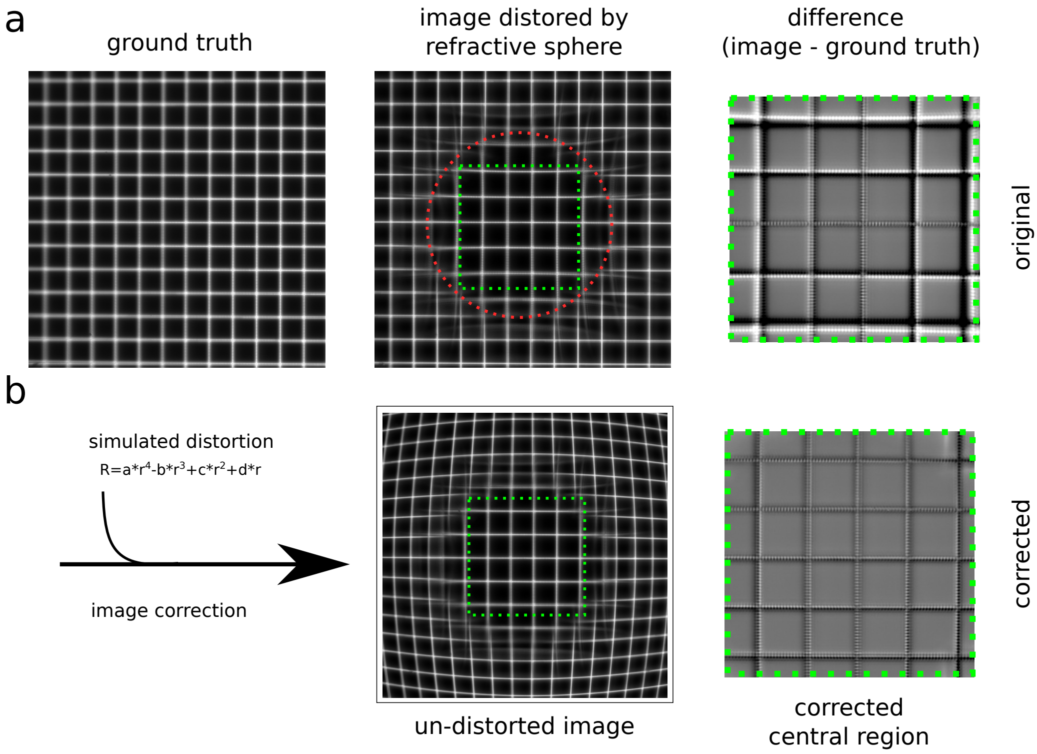

Supplementary Figure 10: a) A rectilinear stripe pattern occurs distorted when imaged through a sphere. b) Fitting a radial distortion map to simulation results of this scenario, allows one to fix the aberrations at the region of interest (green) as seen through the spherical cell phantom.

Supplementary Notes

1 Numerical methods **9**

2 Software implementation & practical use of *biobeam* **11**

3 Examples of *biobeam* programs **13**

4 Numerical validation **17**

5 Experimental validation **18**

6 Performance and comparison with existing software **20**

7 Wave optical forward model in light-sheet microscopy **21**

8 Image-formation in light-sheet microscopy **22**

9 PSF calculations, single and multiplexed **23**

10 Multiplexed aberration calculations **23**

11 Details of memory effect simulations **24**

1 Numerical methods

At the lowest level *biobeam* currently uses the well described scalar beam propagation method (BPM [3, 4]) along with locally reduced refractive index contrasts and a mathematically exact propagator whose use we explain and justify in the following:

The simulation of light propagation through tissue amounts to solving for the electrical field $\vec{E}(x,y,z)$ given a refractive index distribution $n(x,y,z)$ and certain boundary conditions. This in general requires the numerical treatment of the time dependent vectorial *Maxwell's equations*[5]. For monochromatic illumination along z and low refractive index differences however, a far simpler description in terms of a complex scalar field $u(x,y,z)$ becomes applicable, and the problem reduces to solving the scalar

Helmholtz equation[5]:

$$\Delta u(\mathbf{r}) + n(\mathbf{r})^2 k_0^2 u(\mathbf{r}) = 0, \qquad k_0 = \frac{2\pi}{\lambda} \tag{1.1}$$

This approximation exploits the fact that refractive index variations in biological cells are small and light scattering in tissues is predominantly forward directed [2, 6], and that forward directed light scattering determines imaging aberrations. Although back-scattering of light on the way from the specimen to the lens is neglected, this is justified for simulation of the image formation process in tissues, as *i)* this light would only contribute to the final image when changing direction a second time, *ii)* also adaptive optics aberration correction requires the forward scattered photons only.

Eq. (1.1) can be now solved in the spectral or angular spectrum domain [7] by propagating the field $u(x, y, z)$ at position z to $z + \Delta z$ via

$$u(x, y, z + \Delta z) = \int dk_x dk_y \mathcal{F}[u] H(x, y, z, k_x, k_y) e^{i(k_x x + k_y y)} \tag{1.2}$$

where $\mathcal{F}[u](x, y, z) = \int dk_x dk_y u(k_x, k_y, z) e^{-i(k_x x + k_y y)}$ is the 2D Fourier transform of u and $H = e^{i\Delta z \sqrt{n(x,y,z)^2 k_0^2 - k_x^2 - k_y^2}}$ is the accurate (i.e. non paraxial) propagator in the Fourier domain. In a further approximation the refractive index is assumed to be a small variation around a constant $n(x, y, z) = n_0(z) + \Delta n(x, y, z)$ so that the final approximation gives the scalar beam propagation

$$u(x, y, z + \Delta z) \approx \mathcal{F}^{-1}\left[\mathcal{F}[u(x, y, z)] e^{i\Delta z \sqrt{n_0(z)^2 k_0^2 - k_x^2 - k_y^2}}\right] e^{i\Delta z \Delta n k_0} \tag{1.3}$$

which can be efficiently solved by operator split stepping via FFTs and point wise multiplications [3, 4].

To simulate the actual light propagation, we first assume the tissue to be given by a complex grid of refractive indices n(r) after which we employ the classical scalar Fourier transform beam propagation method (FFT-BPM [4]) which solves the scalar Helmholtz equation by a operator split stepping, i.e. first freely propagating the complex field on a given z-plane and then multiplying it by the complex phase difference of the tissue.

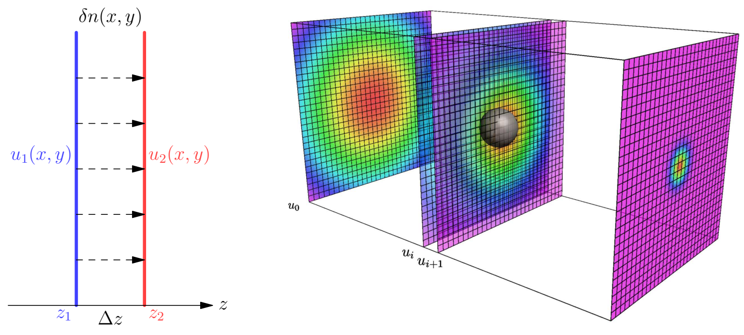

Figure 1: Principle of beam propagation: each z-plane is first propagated freely and then a space dependent phase shift is multiplied.

Crucial for the performance of BPM is a good choice of the refractive index representation $n_0(z)$ while propagating the fields because BPM otherwise quickly becomes inaccurate for non-paraxial light fields. The problems one may encounter are twofold: i) Phase shifts at large angles are under-represented

when refractive index contrasts are high, since the increasing path length with angle is not represented. ii) Inaccuracy could also be introduced in the propagator if the implementation does not match the refractive index representation to calculate phase shifts. To minimize inaccuracies for high angles in regions of large refractive index contrasts, the following representations increase accuracy stepwise:

(a) constant: sets $n_0 = \text{const}$, typically $n_0 = \langle n \rangle_{x,y,z}$, i.e. to the full average of n. This reduces the average refractive index of tissues from typical values 1.37 to 1, thus the remaining refractive index contrast to deal with are just the relative variations that occur in a tissue, typically on the order of $\pm 0.03\%$.
(b) full average: sets $n_0(z) = \langle n \rangle_{x,y}$, i.e. to the per-plane average of n. This bears two advantages. 1) It effectively allows the modeling of aberrations that occur at planar interfaces of high refractive index contrast, i.e. a cover glass or implications of a mismatched working distance. 2) Biological tissues often possess a planar stratification, which implies plane wise varying average refractive indices that can be dealt with more efficiently.
(c) weighted average: sets $n_0(z) = \langle |u|n \rangle_{x,y} / \langle |u| \rangle_{x,y}$, i.e. to the per-plane average weighted by the field magnitude. This choice is often better since, additionally to stratification averages, it accounts for finite lateral dimensions of samples like embryos, i.e. the refractive index for the calculation of the average is only taken into account when the light field is factually overlapping with it.

Already the 1st strategy quoted here largely reduces the problem of inaccuracy of phase projections of high refractive indices at large angles. The two additional tweaks are not generally applicable to any physical light scattering problem, but are of significant practical relevance when dealing with biological samples.

Furthermore, since each algorithmic step is executable in a parallel fashion this method is therefore highly receptive to the parallelization by architecture of modern Graphic Processor units (GPUs). It can be used to calculate the propagation of light through any low contrast refractive index distribution. By this *biobeam* leverages on the enhanced computing power of modern GPUs and attain a significant performance gain compared to a pure CPU implementation (see Section 3).

2 Software implementation & practical use of *biobeam*

The method was implemented within the open source Python[1] software package *biobeam* using OpenCL[2] as the GPU programming architecture. As the computationally heavy parts are lifted to the GPU, we thus keep all the advantages of Python as a dynamically typed high level language that is vastly used in the scientific community without compromising on performance. We chose OpenCL for its availability on all major GPU platforms (NVIDIA, AMD, Intel) and make use of the excellent Python bindings provided by PyOpenCL[3][8]. We further make use of our own utility libraries gputools[4] (for GPU based FFTs, fast spatially varying convolutions, etc) and *spimagine*[5] (rendering/visualization).

[1] Python Software Foundation, http://python.org
[2] Khronos Group, http://khronos.org/opencl
[3] `https://documen.tician.de/pyopencl/`
[4] `https://github.com/maweigert/gputools`
[5] `https://github.com/maweigert/spimagine`

Apart from its technical focus on speed, *biobeam* is specifically designed to make wave optical experiments in-silico as easy as possible. As an example, the listing 2 shows how to propagate a Bessel beam with the annulus defined by the apertures $NA_1 = 0.4, NA_2 = 0.43$ and focal point $50\mu m$ through a random refractive index volume (n $= 1.33 \pm 0.05$) of size $(100\mu m)^3$ on a grid volume $(512, 512, 512)$ and returning the complete complex field on the grid.

biobeam's API further makes it easy to apply different input fields as well as PSF/aberration calculations by propagating diffraction limited point sources from within the tissue. Examples and detailed documentations can be found on at https://maweigert.github.io/biobeam/.

Input fields *Biobeam* offers the possibility to define input fields not only according to the Fourier transform of the aperture function $P(\theta, \phi)$, but more accurately according to the vectorial Debye-Wolf integral (just the x component is shown for brevity, see [9]):

$$E_x(\rho, \varphi, z) = -\frac{ikfE_x^0}{4\pi} \int_0^{\alpha} \int_0^{2\pi} d\theta d\phi \, P(\theta, \phi) \sqrt{\cos\theta} \sin\theta \big[(\cos\theta + 1) + (\cos\theta - 1)\cos 2\phi)\big] \cdot e^{ik\rho \sin\theta \cos(\phi - \varphi)} e^{ikz\cos\theta} \quad (2.1)$$

Cylindrical sheet	Gaussian beam	Bessel beam	Bessel Lattice [10]
`u0_cylindrical(NA=.3)`	`u0_beam(NA=.6)`	`u0_beam(NA=(.3,.32))`	`u0_lattice()`

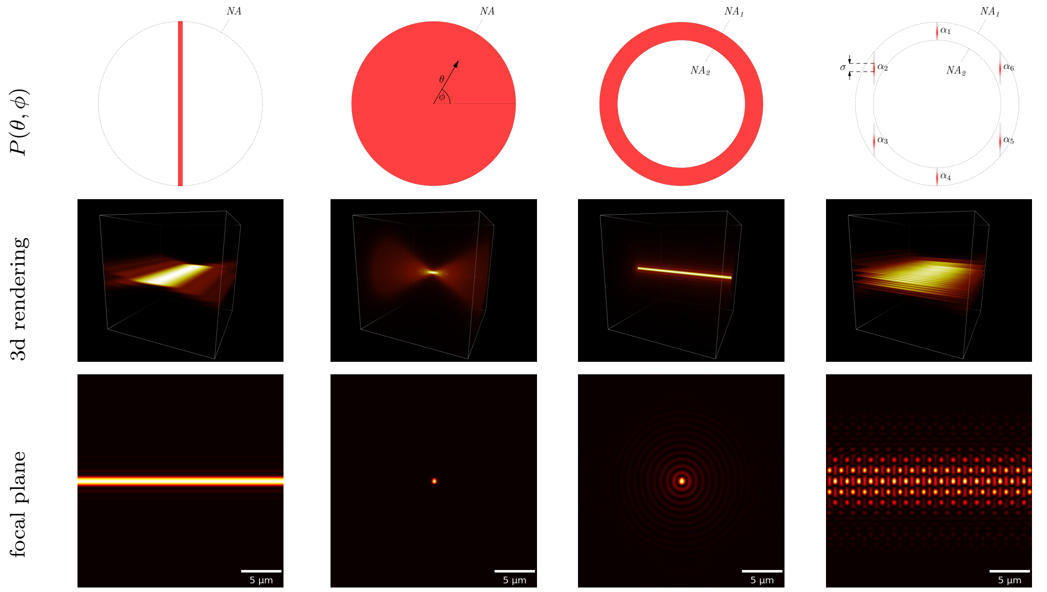

Table 1: Different pupil functions and the resulting focus fields as generated by *biobeam*

Biobeam uses the correctly calculated E_x as input field for the different illumination modes. For that reason the package implements the fast GPU based calculation of these types of volumetric diffraction integrals and PSFs. See listing 1 for a simple example for a Bessel beam.

3 Examples of *biobeam* programs

In this section we give some explicit examples how easy it is to set up wave optical simulations with *biobeam*.

Listing 1: Focussing a Gaussian beam in free space

```
from biobeam import focus_field_beam

# a Gaussian beam with NA = 0.4
intensity = focus_field_beam(
                    shape = (256,256,256),
                    units = (0.1,0.1, 0.1),
                    NA = 0.4)
```

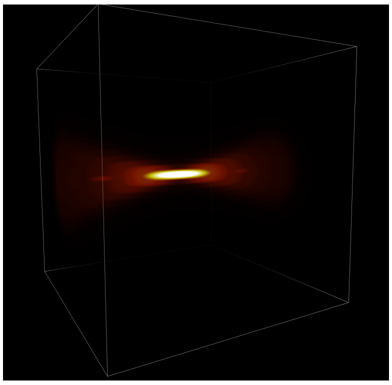

Listing 2: Propagation of a Bessel beam through a tissue phantom, which is given by a Perlin noise refractive index distribution

```
from biobeam import Bpm3d
from gputools import perlin3

# set up the refractive index distribution
dn   = 0.03*perlin3((512,512,512),scale = 4)

# set up the propagator class
m = Bpm3d(dn = dn, size = (70,70,70),
          lam = .5,n0 = 1.33)
# propagate the light field...
field = m.propagate(u0 =
            m.u0_beam(NA = (0.4,.41)))
```

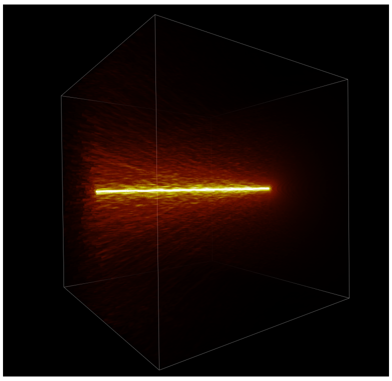

Listing 3: Memory effect: Propagation of a wavefront through scattering medium. Conjugating, shifting and refocusing

```
import numpy as np
from biobeam import Bpm3d

from gputools import perlin3

# set up the refractive index distribution
dn   = 0.03*perlin3((512,512,512),scale = 8)

# set up the propagator class
m = Bpm3d(dn = dn, size = (70,70,70),
          lam = .5,n0 = 1.33)
# propagate a point source through the volume
field0 = m.propagate(u0 =
m.u0_beam(NA = .4, zfoc = 0), return_shape ="last")
#reset r.i.
m2 = Bpm3d(dn = dn[::-1].copy(), size = (70,70,70),
          lam = .5,n0 = 1.33)

# refocus
field1 = m2.propagate(u0 = field0.conjugate(),
return_shape = "last")
# refocus shifted
field2 = m2.propagate(u0 = np.roll(field0.conjugate()
,10,1), return_shape = "last")
```

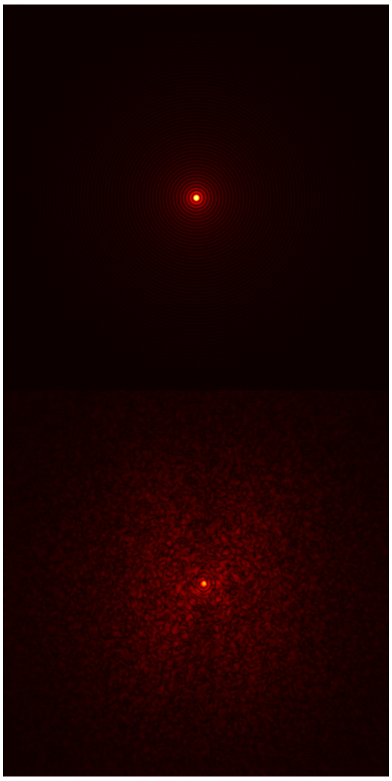

Listing 4: Calculation of the spatially varying PSFs at a plane inside of a refractive sphere

```python
import numpy as np
from biobeam import SimLSM_Cylindrical

def create_dn(N = 512):
        """generates a refractive sphere"""
    x = np.linspace(-50,50,N)
    Xs = np.meshgrid(x,x,x,indexing = "ij")
    R = np.sqrt(np.sum([_X**2 for _X in Xs],
        axis = 0))
    #generate the refractive index differences
    dn = .04*(R<20)
    return dn

dn = create_dn()

#create a microscope simulator
m = SimLSM_Cylindrical(dn=dn,
             NA_illum=.1,NA_detect=.6,
             size = (100,100,100), n0 = 1.33)

# simulate the psf grid at an axial position
# -20um relative to center
psfs = m.psf_grid_z(cz=-20, grid_dim=(16,16),
                    with_sheet = False)
```

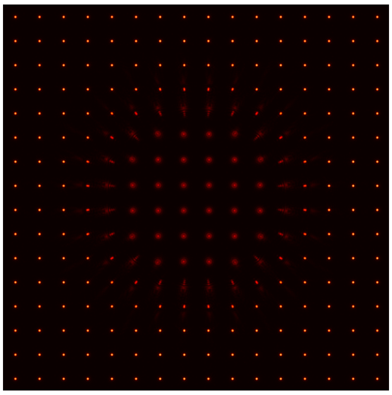

Listing 5: Simulating the image formation of a light-sheet microscope in the presence of a tissue phantom represented by a inhomogeneous refractive sphere of $70\mu m$ diameter. Light sheet in image plane is entering from the left. Time to run should be less than 5 seconds.

```
import numpy as np
from biobeam import SimLSM_Cylindrical
from biobeam.data import tiling
from gputools import perlin3

def create_dn_and_signal(N = 512):
    """generates a refractive inhomogeneous
    sphere and an image as given by the
    function tiling()"""
    x = np.linspace(-50,50,N)
    Xs = np.meshgrid(x,x,x,indexing = "ij")
    R = np.sqrt(np.sum([_X**2 for _X in Xs],
                         axis = 0))
    #generate the refractive index differences
    dn = (.07+0.02*perlin3((N,N,N), scale =3))\
                         *(R<35)
    #replace call to tiling() with own image
    signal = np.einsum("i,jk",np.ones(N),
                         tiling(N))
    return dn, signal

dn, signal = create_dn_and_signal()

#create a microscope simulator
m = SimLSM_Cylindrical(dn=dn, signal=signal,
               NA_illum=.1, NA_detect=.45,
               size = (100,100,100), n0=1.33)

# generate image as recorded by the microscope
# at an axial position -20um relative to center
image = m.simulate_image_z(cz=-20,
psf_grid_dim=(16,16),conv_sub_blocks=(2,2))[16]
```

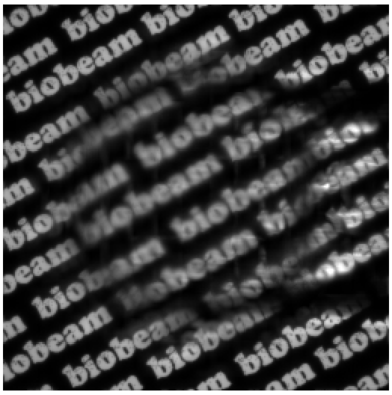

Listing 6: Simulating a light sheet passing through a refractive sample

```
from biobeam import SimLSM_Cylindrical

#create some input data, r.i and labeled density
dn, signal = generate_some_refractive_volume_and_label()

#create a microscope simulator
m = SimLSM_Cylindrical(dn = dn, signal = signal,
                         NA_illum= .1, NA_detect=.6,
                         size = size, n0 = 1.33)
```

```
#simulate the image at relative  axial position 20um
image = m.simulate_image_z(cz=20)
```

Listing 7: Calculating the psf grid at a specific axial position

```
from biobeam import SimLSM_Cylindrical

#create some input data, r.i and labeled density
dn, signal = generate_some_refractive_volume_and_label()

#create a microscope simulator
m = SimLSM_Cylindrical(dn = dn, signal = signal,
                       NA_illum= .2, NA_detect=.6,
                       size = size, n0 = 1.33)

#simulate the image at realtive axial position 20um
psfs = m.psf_grid_z(cz=20, grid_dim=(32,32))
```

4 Numerical validation

To validate the accuracy of the described BMP implementation we compared numerically obtained field distributions with analytically tractable models. Specifically we analyzed the case of incident plane wave scattered by spheres of diameter $5\mu m$ and with a refractive index contrast of 5% as calculated by *biobeam* with the precise mathematical solution (calculated with the Mie code GMMFIELD[6][11]).

We further calculate the scattering phase function $f(\theta,\phi)$ and scattering cross section σ from the angular spectrum $\tilde{U}(k_x,k_y,z_{last})$ of the last plane via

$$\begin{aligned} f(\theta,\phi) &= -ik_0\cos\theta\tilde{U}(k_0\cos\phi\sin\theta, k_0\sin\phi\sin\theta) \\ \sigma &= \int dk_x dk_y |\tilde{U}(k_x,k_y)|^2\sqrt{1-k_x^2/k_0^2-k_y^2/k_0^2} \end{aligned} \tag{4.1}$$

For a concentrically coated sphere as an analytically tractable cell model, with a cytoplasmic fraction and higher refractive cell nucleus, the results are near identical (Supp. Fig.1 c) in both near and far field. Significant relative errors only arise at high angles (see phase function comparison in Supp. Fig. 1d upper graph) at which however little intensity is scattered. We further calculated the scattering efficiencies for coated spheres with various size parameters and compared them to analytical results showing good agreement (see scattering efficiency comparison in Supp. Fig. 1d lower graph). Relative field errors in the analytically limiting case of 3 spheres are still below 1% evaluated over the near-field over a $35\mu m$ big computational cell and all angles (refractive index contrast 3%).

[6] http://moritz-ringler.name/dissertation/GMM_FIELD.tar.bz2

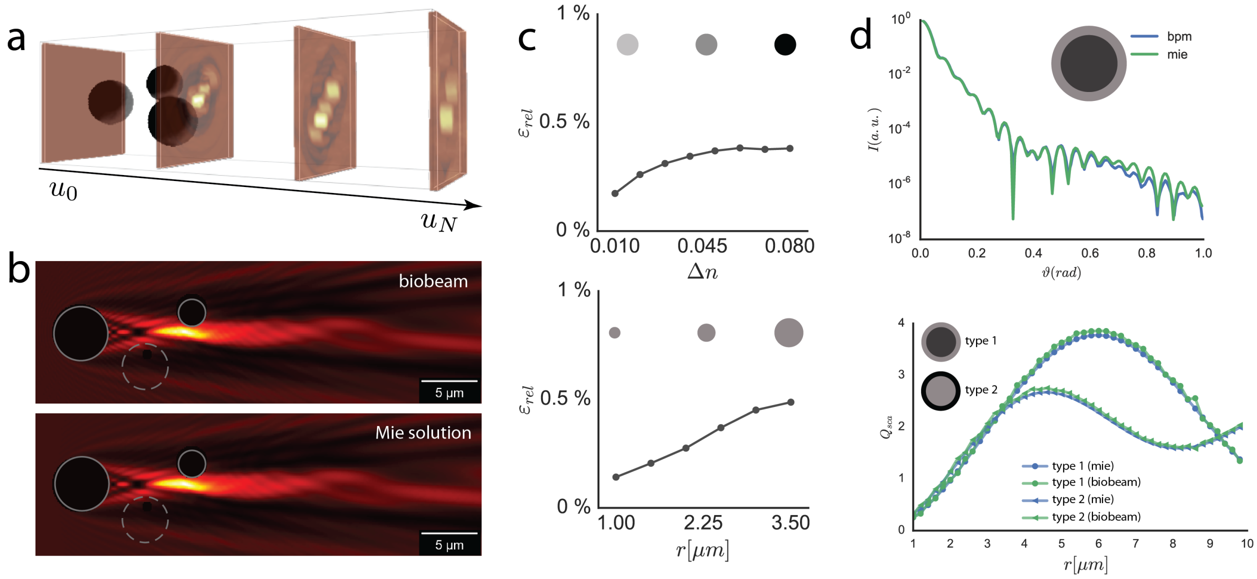

Figure 2: Validation of *biobeam* with analytical solutions. a) Plane wave scattered by three solid spheres (λ=500nm, r=2-2.5μm, refractive index contrast m=1.05), b) Comparison of analytical solution (Mie calculus) versus *biobeam* simulation. c) Error percentage of near field distribution as a function of single sphere radius r (Δn = 0.05) and refractive index contrast Δn (r=2.5μm). d) Top: Phase function of analytically tractable coated spheres as cell models (m=1.02/1.04, r=5μm/4μm) shows high accuracy up to approximately 0.5 radians. bottom: size dependent scattering efficiency of the same sphere architecture and its inverse.

5 Experimental validation

To test the applicability and correctness of *biobeam* in settings relevant for microscopy, we designed a number of imaging experiments and compared them with their reconstitution *in-silico*. Specifically:

- We build a dedicated optical micro-projection setup (Supp. Fig. 7) to record the projected image of a test-chart as seen through refracting spheres and simulated the whole corresponding image formation process *in-silico*.
- We acquired the light intensity behind a diffracting edge and compared it with the results obtained with *biobeam*.
- We measured the light scattered from agarose embedded spheres in a commercial light-sheet microscope and compared it with the simulation pipeline.

Image micro-projection through spheres

Experimental setup: Experiments were performed using a custom micro projection set up built in our lab (Supp. Fig. 7), controlled with custom LabView programs. The set up allows for patterns to be micro projected on to a sample with predefined illumination-source, size, magnification and NA of influx optics. The efflux optics allows for the collection of the light and be recorded on the camera. A negative USAF (R1DS1N, Thorlabs) chart was illuminated incoherently (M470L3 Thorlabs) and the resolution grids were projected on glass spheres (Borosilicate material, $n = 1.48$, 110μm diameter,

Cospheric LLC, USA) embedded in OptiPrep ($n = 1.42$). The influx and efflux objectives were, 20x/0.45 NPL Fluotar P Leitz-Wetzlar, Germany and Olympus UPLSAPO 20x/0.75 respectively. The images were captured using a Andor Zyla 5.5 sCMOS camera.

Simulation: A computational grid of size $(1024, 1024, 730)$ with a pixel size of $\Delta x = 0.161\mu m$ was used and a spherical refractive index distribution with $n = 1.48, r = 110\mu m$ was defined, using $n = 1.42$ as the refractive index of the outer medium). We then calculated a 16×16 grid of detection PSFs corresponding to an imaging plane directly behind the sphere. To ensure non overlapping signal of the individual PSFs, we multiplexed the PSF simulation according to a 2×2 checkerboard pattern. Finally, we performed a spatially varying convolution of the input (test-chart) image with the obtained PFSs.

Diffraction around a knife edge

Experimental setup: The Thorlabs R1L3S6P variable line grating test chart, with one of the 1.25lpm grating was used as an edge. The light was focused with an incoherent light source (M470L3 Thorlabs) such that a pseudo plane wave ($NA = 0.001$) illuminated the edge. The diffracting light was imaged below at different depths from the edge.

Simulation: A computational grid of size $(1024, 256, 1830)$ with a pixel size of $\Delta x = 0.29\mu m$ was used. As incident light distribution at the knife edge, we used a plane wave

$$u_0(x, y) = e^{2\pi\lambda k_x x}\theta(x - x_0)$$

clipped at the position of the edge x_0. For the coherent case we set $\lambda = 0.47\mu m$, for the incoherent case we uniformly sample $\lambda \sim \mathcal{U}(0.46\mu m, 0.48\mu m)$ and averaged the intensities of 100 such sampled plane waves, thus taking into account the spectral width of the illumination source ($\pm 10nm$, from measured spectra).

Light sheet imaging experiments

Experimental setup: The light sheet experiments were performed on a Light-sheet Z.1 microscope (Zeiss, Germany) equipped with a Clr Plan-Neofluar 20x/1.0 detection objective corrected for a refractive index of $n = 1.42 - 1.48$ and two 10x/0.2 illumination objectives. Both Pivot Scanner and Light-sheet Scanner were deactivated to obtain a stationary light sheet with a waist of $1.7\mu m$ and a lateral width appr. $100\mu m$ FWHM. Images were recorded using a PCO.Edge sCMOS camera and a 1.0x zoom lens resulting in a final pixel size of $0.229\mu m \times 0.229\mu m$. Stacks were acquired at a step size of $0.414\mu m$. Alexa Fluor 488 was excited at $488nm$ and detected with a $505 - 545nm$ bandpass filter. Polymethylmethacrylate (PMMA) microparticles ($n = 1.495$) with a diameter of $20\mu m$ (PolyAn GmbH, Germany) were mounted in an agarose block and immersed in OptiPrep (Progen Biotechnik GmbH, Germany) with a refractive index of $n \sim 1.429$. Blocks were prepared from low gelling agarose (Sigma Aldrich, USA) in OptiPrep at a concentration of 1.0%, labelled with Alexa Fluor 488 carboxylic

acid ($5\mu g/ml$, Thermo Fisher Scientific, USA) and mixed with the PMMA microparticles at a ratio of $1:4,000$ ($10\mu l$ of 5% stock solution in 2 ml agarose).

Simulation: A computational grid of size $(1024, 512, 512)$ with a pixel size of $\Delta x = 0.205\mu m$ was used and a spherical refractive index distribution with $n = 1.495, r = 10\mu m$ was defined at the position given by the experimental image stack. As illumination field, a light sheet was simulated with $NA = .2$ and $\lambda = 0.49\mu m$ incident on the sphere. Next a grid of (16×16) detection PSFs was generated (detection $\lambda = 0.52$)) and convolved (spatially varyingly) with the illumination intensity to give the final image.

Outlook on distortion and aberration correction

As a very simple example of how simulated aberrations can be helpful to correct experimental distortions we consider the case of the distorted image of a stripe pattern projected through a refracting sphere. The 2D grid patterns were generated using a custom LabView program and micro projected as before through a borosilicate glass microspheres (RI = 1.48) of 110um diameter with Iodixanol-Water solution (OptiPrep, RI = 1.429 confirmed by Abbe refractometry) acting a surrounding media. Based on the 2D grid pattern simulation (as before) of the experimental setting a 4th degree polynomial lens radial distortion model was fitted and the experimentally recorded data undistorted by the open-source image processing suite Hugin (http://hugin.sourceforge.net/docs/manual/Lens_correction_model.html). See Supplemental Fig. 10 for the results.

6 Performance and comparison with existing software

We compared the attainable performance of *biobeam* with available open source programs, specifically with MEEP[12][7], which implements the Finite Difference Time Domain (FDTD) method to solve Maxwells' equation and the Mie code GMMFIELD[11] for internal field calculations.

Dimension ($x \times y \times z$)	Mie code (GMMFIELD)	FDTD (MEEP)	*biobeam* BPM
(128,128,128)	1314s	80 s	34 ms
(256,256,256)	10480s	790 s	81 ms
(512,512,512)	–	4800 s	154 ms
(1024,1024,1024)	–	–	440 ms

Table 2: Runtimes of plane wave propagation through a given refractive index distribution of given dimensions

The runtime differences of several orders of magnitude clearly demonstrates the superiority of the BPM as implemented on GPUs. This efficiency results from a scaling of operations directly proportional to

[7] http://ab-initio.mit.edu/wiki/index.php/Meep

the penetration depth into the tissue compared to Mie and FDTD codes, and a further speed up due to the massive parallelization of remaining operations on the GPU.

To measure the speedup of the GPU/OpenCL based implementation compared to a pure CPU (in C) and GPU/CUDA implementation we compared the runtime of a plane wave propagating through a constant refractive index distribution for three different basic implementations of the BPM method.

CPU Single precision implementation in C (FFTW 3.3 with `FFTW_MEASURE` as FFT library), and different levels of multithreading with 1, 4 or 16 threads.

GPU1 Python with PyCuda bindings

GPU2 Python with PyOpenCL bindings

The hardware used was a 20 core Xeon(R) CPU E5-2660 v3 (2.60GHz, 64GB RAM) workstation for the CPU benchmarks, and a NVIDIA GeForce GTX Titan X (12GB RAM) for the GPU ones.

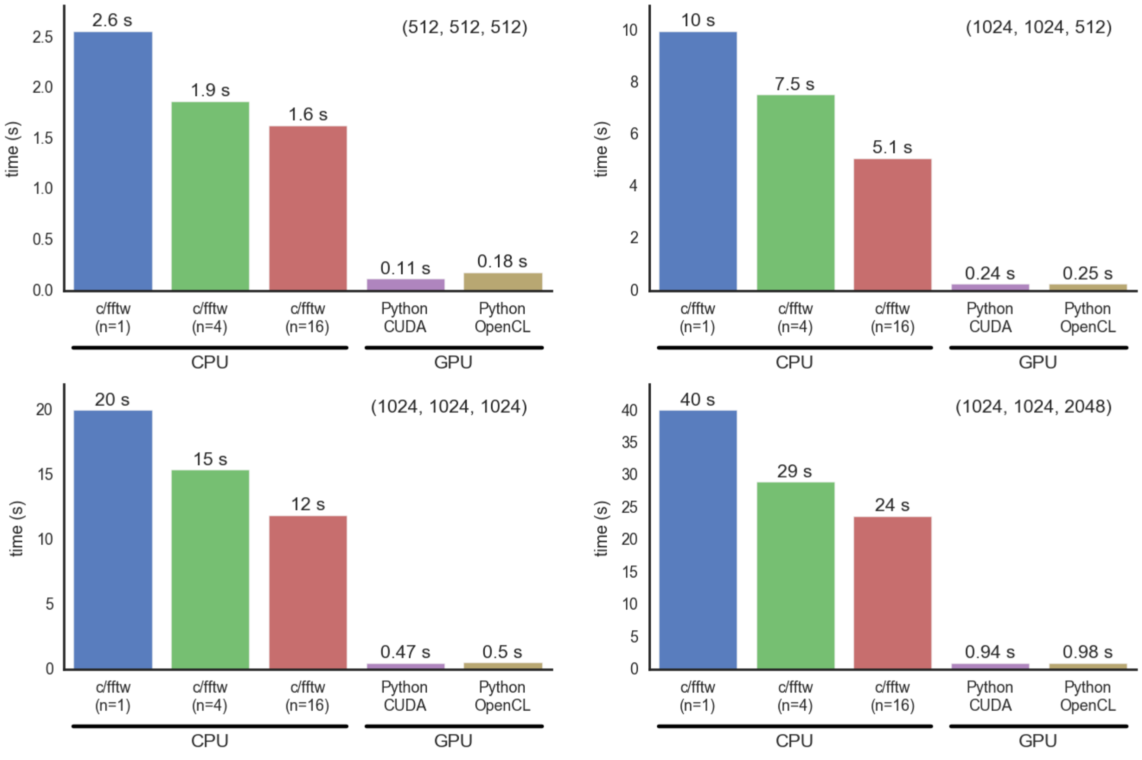

Figure 3: Comparison for different BPM implementations on CPU vs GPU

The performance of both the OpenCL and CUDA implementation are compareable and the overall speedup when of the OpenCL version compared to the single threaded CPU version was between 15–40 and to the 16-multithreaded version between 10–25, where the greater speedups were achieved in the relevant scenario of bigger propagation volumes.

7 Wave optical forward model in light-sheet microscopy

Biobeam is a modular software suite to enable the simulation of the image formation process in tissue microscopy. While the modularity of our software enables to flexibly implement the simulation of dif-

ferent imaging modalities, we exemplify *biobeam*'s performance by the implementation of a light-sheet microscope, which makes use of two distinct light paths, one for excitation and one for the collection of fluorescent light. We start this section with a general description of the process and complement this by the detailed explanation of how *biobeam* efficiently carries out the actual simulations required to faithfully mimic the microscopy process.

8 Image-formation in light-sheet microscopy

Light-sheet microscopy achieves optical sectioning by the orthogonality between the illumination and the detection axis [13]. To model the image-formation process let

$f(x,y,z)$, the fluorophore density in the sample
$h(x,y,z)$, the PSF of the detection objective
$j(x,y,z)$, the illumination intensity (PSF) of the light sheet
$g(x,y,z)$, the observed volumetric intensity (image)

where z is the coordinate along the optical axis of the detection objective and y the propagation axis of the illuminating field. Let the current focal position be z_F and fixed. Then the excited intensity distribution of the fluorophore $\tilde{f}(x,y,z)$ is

$$\tilde{f}(x,y,z) = f(x,y,z) \cdot j(x,y,z_F - z) \tag{8.1}$$

For each constant slice at z the intensity $\tilde{f}(x,y,z)$ gets convolved with $h(x,y,z_F - z)$ resulting in

$$a(x,y,z_F,z) = \int dx'dy' h(x',y',z_F - z)\tilde{f}(x-x',y-y',z) \tag{8.2}$$

$$= \int dx'dy' h(x',y',z_F - z) f(x-x',y-y',z) j(x-x',y-y',z_F - z) \tag{8.3}$$

and the overall observed image plane $g(x,y,z_F)$ for a fixed focal position is then the integral of all contributions

$$g(x,y,z_F) = \int dx'dy'dz' h(x',y',z') f(x-x',y-y',z_F - z') j(x-x',y-y',z') \tag{8.4}$$

Note that if the illumination field can be factored as $j(x,y,z) = j_{xy}(x,y) \cdot j_z(z)$, then

$$g(x,y,z_F) = (h \cdot j_z) \otimes (f \cdot j_{xy}) \tag{8.5}$$

and the observed image g is simply the 3 dimensional convolution of the illuminated density $f \cdot j_{xy}$ with an effective PSF $h_{eff} = h \cdot j_z$. For a distorted illumination field, this however is not true in general and the detection PSF as well typically varies spatially due to the distortions induced by the tissue. For modeling this complete image-formation process *biobeam* executes the following steps at every desired axial position z_F (cf. Video 2):

1. computing the distorted illumination field j at a given position z_F

2. calculating a fine grid of multiplexed spatially varying detection PSFs at that axial position, and
3. performing with it a spatially varying convolution of the product of the fluorophore signal and the 3d excitation profile to produce the final image at z_F as seen by the detector.

For 1. the resulting PSFs are interpolated between sampling points to result in a quasi-continuum that can be used for an accurate convolution with a spatially varying, wave-optically determined kernel. The latter step is carried out based on standard methods [14]. The API of *biobeam* allows for a simple way of doing these image-formation simulations, as demonstrated by the listing 5 for the case of a cylindrical light-sheet microscope:

9 PSF calculations, single and multiplexed

Single PSF Point spread functions inside tissues were calculated by propagating light from tissue embedded, diffraction-limited airy disks corresponding to the given numerical aperture (NA) of the detection system towards an idealized lens. Here the resulting complex wave field is phase-conjugated and propagated back towards a focus, but this time through free space (cf. Video 2). For light-sheet microscopy simulations these point spread function are significantly further constrained in axial direction due to the small width of the independently simulated light sheet.

Multiplexed PSFs For the parallel simulation of a complete grid of spatially varying PSFs we exploit the fact that optics is linear, and sufficiently spaced point spread functions in a plane do not significantly overlap, hence can be propagated and refocused in multiplexed way (cf. Supp. Fig. 3). The PSF grid sampling is practically done at a grid-spacing below the smallest isoplanatic patch size in the sample, which are found at the most distant positions from the imaging lens, resulting in a sub-sampling of the volume proportional to the half-width of the aberration-autocorrelation function for the plane furthest from the objective. These PSFs are efficiently generated all at once in a multiplexed manner, i.e. a sub-grid of initial diffraction limited focal spots is propagated simultaneously through the volume and refocused together while ensuring that no substantial coherent overlap arises between neighboring PSFs (cf. Supp. Fig. 3 and S3 Video). This reduces the computational complexity by the number of simultaneously calculated PSFs, i.e. typically 100-1000 fold.

Listing 4 demonstrates how one can calculate the grid of point spread function in a highly multiplexed manner on a (32×32) grid at relative axial position 20μ for a cylindrical lens SPIM with illumination $NA_{illum} = 0.2$ and detection $NA_{detect} = 0.5$.

10 Multiplexed aberration calculations

In practice, it might often be desired not only simulate sets of PSFs distributed over a sample, but to directly extract spatial maps of aberrations that would result when imaging a tissue or embryo that is represented in a refractive index model.

Given the efficiently multiplexed determination of PSF as described in the previous section (Supp. Fig. 3), also aberrations terms can efficiently be calculated for any point in a sample in the same manner. The main benefit again is that a single multiplexed simulation through the tissue is sufficient. Let $h(x-x_0, y-y_0, z_F)$ be the lateral slice of the PSF associated to a given initial position (x_0, y_0) at focal position z_F, then the corresponding pupil function $P(\theta, \phi)$ is given by the properly rescaled Fourier transform of $h(x-x_0, y-y_0, z_F)$. Projecting $P(\theta, \phi)$ onto a Zernike basis yields the associated Zernike terms of the aberrations present at (x_0, y_0, z_F). Supp. Fig. 4 shows a demonstration of these aberration calculations for a tissue model with a biological plausible refractive index distribution mimicking cell nuclei, cytoplasm and the eggshell of of $n \in (1.35, 1.43)$ [1].

Biobeam is highly efficient in determining sample induced aberration for given refractive index models (cf. Supp. Fig. 4). For this, light from diffraction limited guide-stars is propagated to the back-pupil of a virtual microscope. Recorded field can then be composed according to Zernike aberration modes. For this we record the complex scalar product between the field distribution and each Zernike mode on a pupil representing unit-sphere.

11 Details of memory effect simulations

The shift-shift memory effect [2] describes the behavior of an aberration corrected and therefore diffraction limited focal spot when laterally translated within a scattering tissue. Given a fixed position g, such focus can be created by pre-shaping the incoming wavefront with the correct wavefront aberration terms ϕ_g [15]. When translated laterally by a length Δx, the quality of the resulting spot at the translated position quickly deteriorates (Video 6), albeit in a charactized manner: The correlation coefficient as a function of Δx between the original and the laterally shifted focal spot is equal to the autocorrelation function of the speckle pattern resulting from an incident plane wave at the same depth [2].

To simulate the memory effect as in Fig. 2, we first created synthetic refractive index distributions at different depth ($N_x/N_y = 100\mu m, N_z = 20 \ldots 80\mu m$) and with refractive index variation of $n = 1.35 \pm 0.03$ by either randomly placing hard spheres in the volume or using generated Perlin noise of the given variation. Next, diffraction limited guide stars were implemented inside this tissue dummy and propagated to its surface, where the guide-star specific aberration patterns ϕ_g were recorded. Phase conjugation of these fields at the tissue surfaces leads to the precise recovery of the initial PSF inside the tissue. Reduction of Strehl ratio was then determined as the average intensity of the focus that is recreated for laterally displaced fields at the tissue surface. The decay of focal intensity with lateral shifts were found identical to the absolute value of the spatial correlation in a speckle field that results from an incident plane-wave. The area of the isoplanatic patch was calculated as the radial integral over the Strehl ratio increase gained from the aberration pre-compensation. This is significantly smaller than the actual Strehl ratio only for small penetration depths (d $\leq$ 1 MFP) at which focusing still works to a residual degree without adaptive optics. For all simulations in Fig. 3c, the average of 50 different guide stars at 150 different translation position were simulated for for the shift-shift memory effect at 3 different depths, comprising 7500 focii calculated per depth and 22500 in total.

References

1. Choi, W. *et al.* *Nature Methods* **4,** 717–719 (2007).
2. Judkewitz, B., Horstmeyer, R., Vellekoop, I. M., Papadopoulos, I. N. & Yang, C. *Nature physics* **11,** 684–689 (2015).
3. Fertig, M. & Brenner, K.-H. *J. Opt. Soc. Am.* **27,** 709–717 (2010).
4. Van Roey, J., Van der Donk, J. & Lagasse, P. *J. Opt. Soc. Am.* **71,** 803–810 (1981).
5. Born, M. & Wolf, E. *Principles of Optics* 7th ed. (Cambridge University Press, 1999).
6. Jacques, S. L. *Physics in Medicine and Biology* **58,** R37 (2013).
7. Goodman, J. *Introduction to Fourier Optics* 2nd ed. (MaGraw-Hill, 1996).
8. Klöckner, A. *et al.* *Parallel Computing* **38,** 157–174. ISSN: 0167-8191 (2012).
9. Foreman, M. R. & Török, P. *Journal of Modern Optics* **58,** 339–364 (2011).
10. Chen, B.-C. *et al.* *Science* **346,** 1257998 (2014).
11. Ringler, M. *Plasmonische Nahfeldresonatoren aus zwei biokonjugierten Goldnanopartikeln* PhD thesis (LMU, 2008).
12. Oskooi, A. F. *et al.* *Computer Physics Communications* **181,** 687 (Mar. 2010).
13. Huisken, J., Swoger, J., Del Bene, F., Wittbrodt, J. & Stelzer, E. H. *Science* **305,** 1007–1009 (2004).
14. Nagy, J. G. & O'Leary, D. P. *SIAM Journal on Scientific Computing* **19,** 1063–1082 (1998).
15. Vellekoop, I. M. & Mosk, A. *Optics Letters* **32,** 2309–2311 (2007).
