# Supplementary material for: Biobeam—Multiplexed wave-optical simulations of light-sheet microscopy
Source: PLoS Comput Biol. 2018 Apr 13;14(4):e1006079. doi: 10.1371/journal.pcbi.1006079 (PMC5898703; doi:10.1371/journal.pcbi.1006079)
Supplement: S1 Fig — a) Plane wave scattered by three solid spheres (λ = 500nm, r = 2-2.5μm, refractive index contrast m = 1.05), b) Comparison of analytical solution (Mie calculus) versus biobeam simulation. c) Error percentage of near field distribution as a function of single sphere radius r (Δn = 0.05) and refractive index contrast Δn (r = 2.5μm). d) Left: Phase function of analytically tractable coated spheres as cell models (m = 1.02/1.04, r = 5μm/4μm) shows high accuracy up to approximately 0.5 radians. Right: size dependent scattering efficiency of the same sphere architecture and its inverse. (PDF) [file pcbi.1006079.s009.pdf]

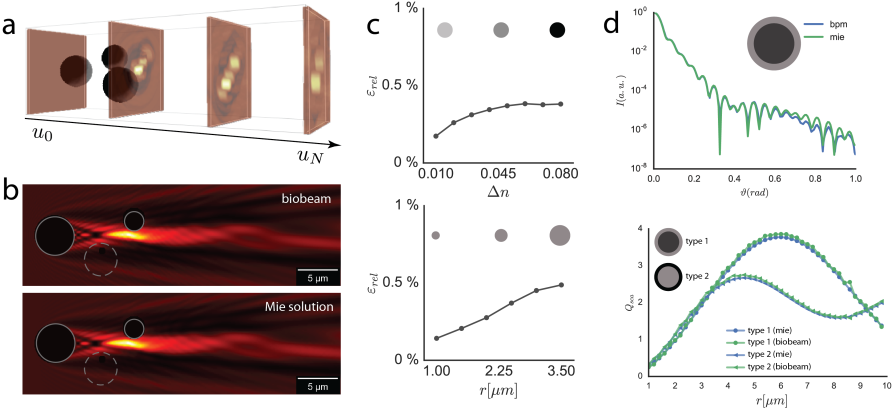

Supplementary Figure 1: Validation of *biobeam* with analytical solutions. a) Plane wave scattered by three solid spheres (λ=500nm, r=2-2.5μm, refractive index contrast m=1.05), b) Comparison of analytical solution (Mie calculus) versus *biobeam* simulation. c) Error percentage of near field distribution as a function of single sphere radius r ($\Delta n = 0.05$) and refractive index contrast Δn (r=2.5μm). d) Top: Phase function of analytically tractable coated spheres as cell models (m=1.02/1.04, r=5μm/4μm) shows high accuracy up to approximately 0.5 radians. Bottom: size dependent scattering efficiency of the same sphere architecture and its inverse.
