# Supplementary material for: Biobeam—Multiplexed wave-optical simulations of light-sheet microscopy
Source: PLoS Comput Biol. 2018 Apr 13;14(4):e1006079. doi: 10.1371/journal.pcbi.1006079 (PMC5898703; doi:10.1371/journal.pcbi.1006079)
Supplement: S2 Fig — The respective pupil function is shown in the upper row. (PDF) [file pcbi.1006079.s010.pdf]

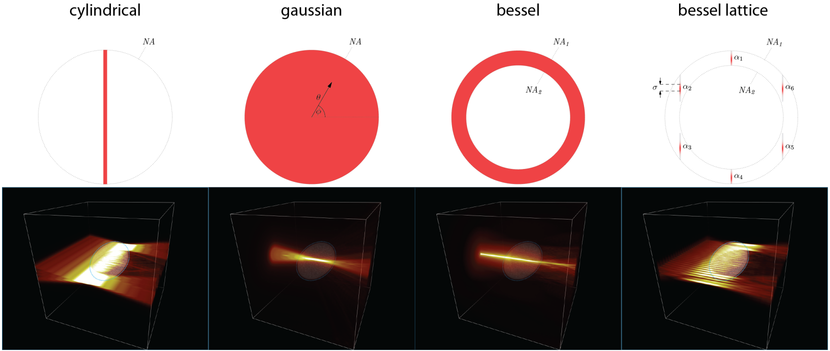

Supplementary Figure 2: Propagation of different predefined input fields through a tissue model of size $(100\mu m, 100\mu m, 100\mu m)$ and grid dimension (1024^3). The respective pupil function is shown in the upper row.
