# Supplementary material for: Biobeam—Multiplexed wave-optical simulations of light-sheet microscopy
Source: PLoS Comput Biol. 2018 Apr 13;14(4):e1006079. doi: 10.1371/journal.pcbi.1006079 (PMC5898703; doi:10.1371/journal.pcbi.1006079)
Supplement: S3 Fig — Propagating a diffraction limited input field through parts of the sample and refocusing by an idealized optical system gives the focus field as seen by the detector. If the refocus spots are separated for different starting points, the propagation of a complete grid can be carried out in a highly multiplexed manner, accelerating the process for typical microscopy simulations by a factor 100–1000. (PDF) [file pcbi.1006079.s011.pdf]

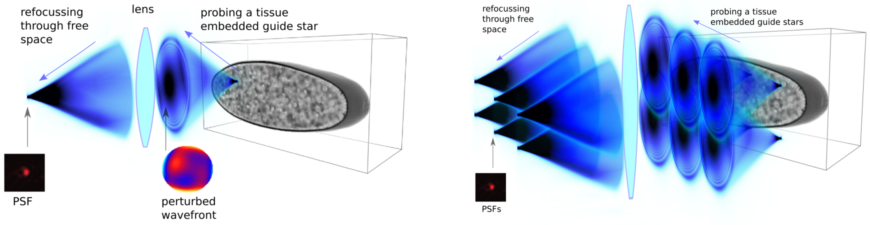

Supplementary Figure 3: Detection aberration and PSF calculation. Propagating a diffraction limited input field through parts of the sample and refocusing by an idealized optical system gives the focus field as seen by the detector. If the refocus spots are separated for different starting points, the propagation of a complete grid can be carried out in a highly multiplexed manner, accelerating the process for typical microscopy simulations by a factor 100–1000.
