# Supplementary material for: Biobeam—Multiplexed wave-optical simulations of light-sheet microscopy
Source: PLoS Comput Biol. 2018 Apr 13;14(4):e1006079. doi: 10.1371/journal.pcbi.1006079 (PMC5898703; doi:10.1371/journal.pcbi.1006079)
Supplement: S4 Fig — The model’s physical size is (200μm, 100μm, 100μm) and the dimension of the computational grid is (1024, 512, 512). The detection wavelength is λ = 522nm, the numerical aperture is NA = 0.5 and the aqueous immersion medium has refractive index n0 = 1.33. The refractive index distribution of the tissue model mimics an eggshell, cell nuclei and granular random fluctuations within the biological plausible range of n ∈ (1.35, 1.43). (PDF) [file pcbi.1006079.s012.pdf]

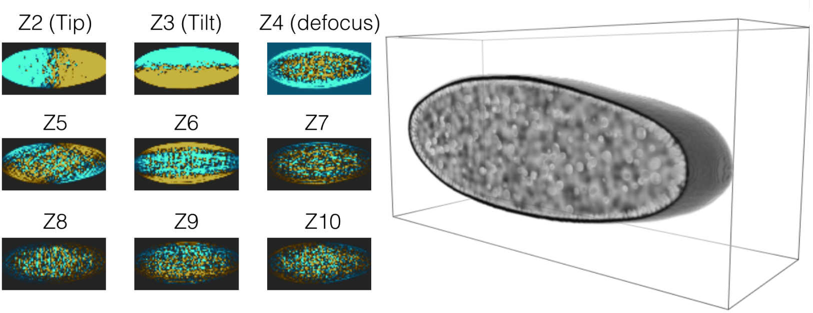

Supplementary Figure 4: Calculating of the aberrations of the detection point spread function (PSF) for a given z plane within a synthetic tissue model. The model's physical size is $(200\mu m, 100\mu m, 100\mu m)$ and the dimensions of the computational grid are $(1024, 512, 512)$. The detection wavelength is $\lambda = 522nm$, the numerical aperture is $NA = 0.5$ and the aqueous immersion medium has a refractive index of $n_0 = 1.33$. The refractive index distribution of the tissue model mimics an eggshell, cell nuclei and granular random fluctuations within the biological plausible range of $n \in (1.35, 1.43)$.
