# Supplementary material for: Biobeam—Multiplexed wave-optical simulations of light-sheet microscopy
Source: PLoS Comput Biol. 2018 Apr 13;14(4):e1006079. doi: 10.1371/journal.pcbi.1006079 (PMC5898703; doi:10.1371/journal.pcbi.1006079)
Supplement: S5 Fig — a) Experimental setup: Light is focused with an incoherent light source (M470L3 Thorlabs, λ = 470nm) such that an almost plane wave (NA = 0.001) illuminated the knife edge. The diffracting light was imaged below at different depths from the edge. b) The simulation was done on a computational cell of size (1024 × 256 × 1830) with voxel size Δx = 0.29μm. We simulated the diffraction in the case of a single plane wave (coherent, top) and the incoherent superposition of 100 random incident plane waves of different small incident angle (corresponding to NA = 0.001, bottom). c) The experimental acquired intensity. Scalebar is 12μm in both axial and lateral direction (depicted with axial/lateral aspect ratio of 8, due to space constraints). d) Intensity plot at a given axial position (dashed line) for simulation, experiment and the intensity calculated via Fresnel-integral (Theory). (PDF) [file pcbi.1006079.s013.pdf]

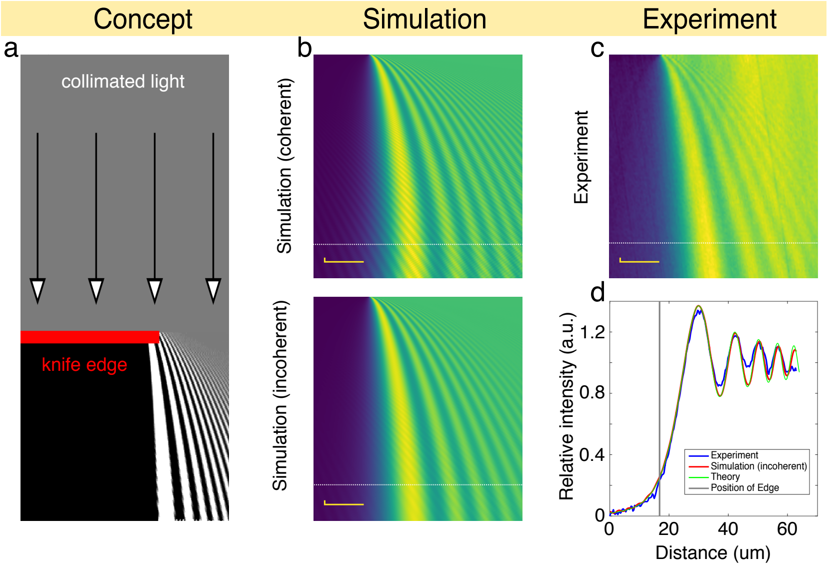


**Supplementary Figure 5:** Diffraction around a knife edge. a) Experimental setup: Light is focused with an incoherent light source (M470L3 Thorlabs, $\lambda_0 = 470nm$) such that an almost plane wave ($NA = 0.001$) illuminated the knife edge. The diffracting light was imaged below at different depths from the edge. b) The simulation was done on a computational cell of size $(1024 \times 256 \times 1830)$ with voxel size $\Delta x = 0.29\mu m$. We simulated the diffraction in the case of a single plane wave (coherent, top) and the incoherent superposition of 100 incident plane waves of uniformly sampled wavelengths $\lambda \in [460nm, 480nm]$, corresponding to the measured spectral width of $\pm 10nm$ of the light source (incoherent, bottom). c) The experimentally acquired intensity. Scale bar is $12\mu m$ in both axial and lateral direction (depicted with axial/lateral aspect ratio of 8, due to space constraints). d) Intensity plot at a given axial position (dashed line) for simulation, experiment and the intensity calculated via Fresnel-integral (Theory).
