# Supplementary material for: Biobeam—Multiplexed wave-optical simulations of light-sheet microscopy
Source: PLoS Comput Biol. 2018 Apr 13;14(4):e1006079. doi: 10.1371/journal.pcbi.1006079 (PMC5898703; doi:10.1371/journal.pcbi.1006079)
Supplement: S6 Fig — a) Poly-methylmethacrylate (PMMA) micro-particles with a diameter of 20μm and refractive index of n = 1.495 were embedded in an OptiPrep/agarose block (n ≈ 1.43) labelled with Alexa Fluor 488. A stationary illuminating light-sheet with a waist of 1.7μm and a lateral extension of ≈100μm was generated with a LZ1 (Zeiss) light-sheet microscope, incident on the agarose embedded spheres. Stacks were acquired at a step size of 0.414μm. b) Simulation results of the intensity distribution behind the sphere at a plane incident to the sphere center. c) Experimental intensity image. Scalebar is 20μm in both cases. Dashed lines indicate regions with specific diffraction patterns that the simulation correctly reconstitutes. (PDF) [file pcbi.1006079.s014.pdf]

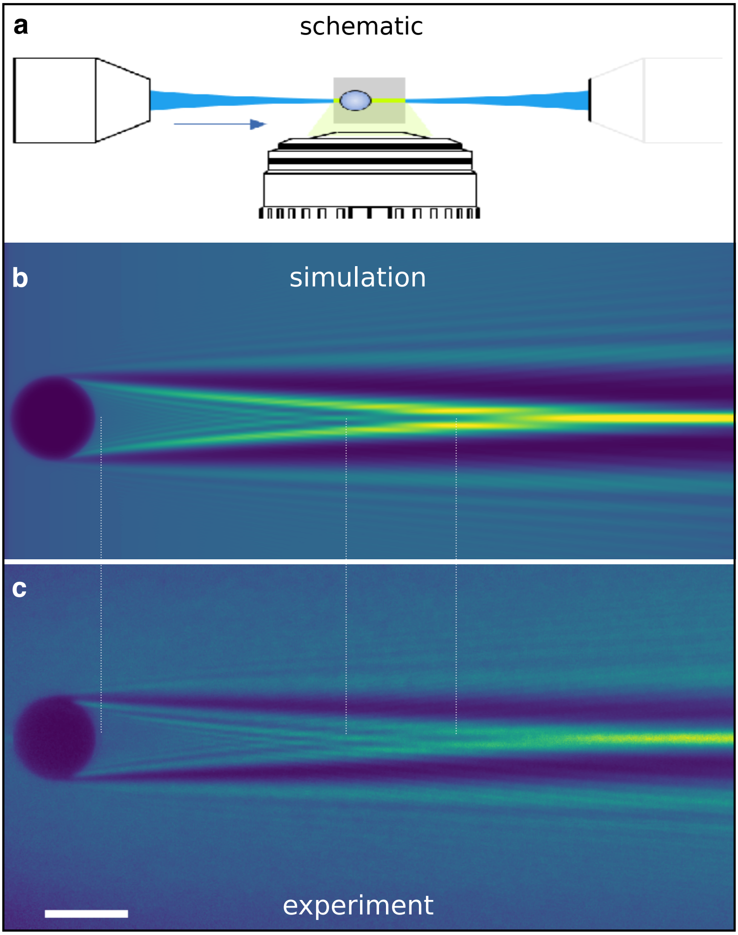

Supplementary Figure 6: Experimental validation on a commercial light-sheet microscope. a) Polymethylmethacrylate (PMMA) microparticles with a diameter of $20\mu m$ and refractive index of $n = 1.495$ were embedded in an block of OptiPrep (Progen Biotechnik GmbH) / agarose (Sigma Aldrich) with refractive index of $n \approx 1.43$) and which was labelled with Alexa Fluor 488. A stationary illuminating light sheet with a waist of $1.7\mu m$ and a lateral extension of $\approx 100\mu m$ was generated with a LZ1 (Zeiss) light-sheet microscope, incident on the agarose embedded sphere. Stacks were acquired at a step size of $0.414\mu m$. b) Simulation results of the intensity distribution behind the sphere at a plane incident to the sphere center. c) Experimental intensity image. Scale bar is $20\mu m$ in both cases. Dashed lines indicate regions with specific diffraction patterns that the simulation correctly reconstitutes.
