# Supplementary material for: Biobeam—Multiplexed wave-optical simulations of light-sheet microscopy
Source: PLoS Comput Biol. 2018 Apr 13;14(4):e1006079. doi: 10.1371/journal.pcbi.1006079 (PMC5898703; doi:10.1371/journal.pcbi.1006079)
Supplement: S7 Fig — The setup allows for patterns to be micro projected onto a sample with predefined illumination-source, size, magnification and NA of influx optics. The efflux optics allows for the collection of the light and recording on the camera. (PDF) [file pcbi.1006079.s015.pdf]

Micro-projection & transmission setup

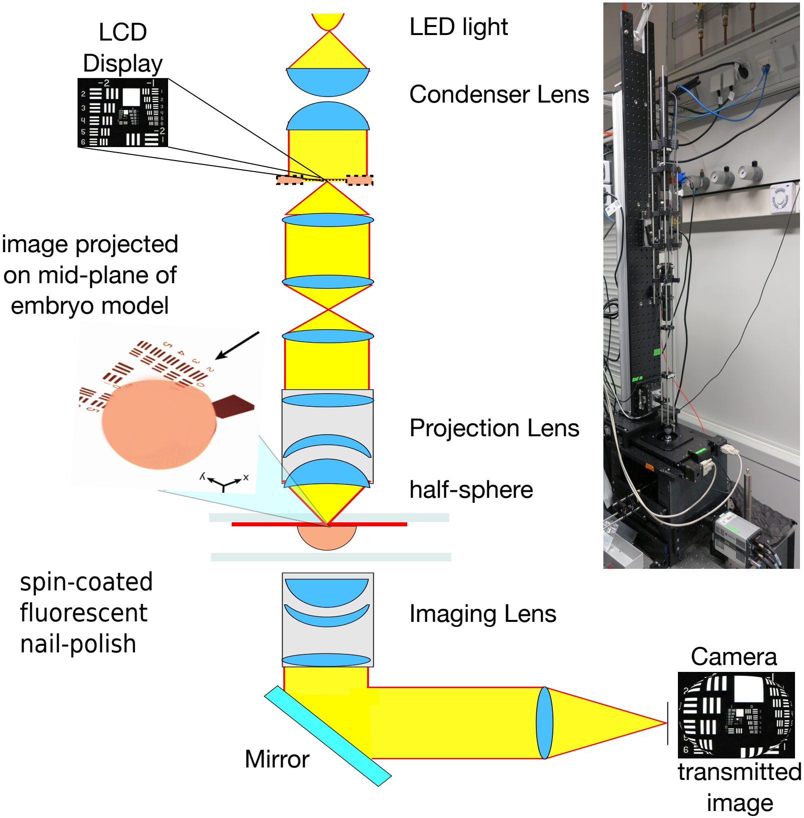

Supplementary Figure 7: Custom micro-projection setup built in our lab, controlled with custom LabView programs. The setup allows for patterns to be micro-projected onto a sample with predefined illumination-source, size, magnification and NA of influx optics. The efflux optics allow for the collection of the light and recording on the camera.
