# Supplementary material for: Biobeam—Multiplexed wave-optical simulations of light-sheet microscopy
Source: PLoS Comput Biol. 2018 Apr 13;14(4):e1006079. doi: 10.1371/journal.pcbi.1006079 (PMC5898703; doi:10.1371/journal.pcbi.1006079)
Supplement: S8 Fig — Experimental setup as in S7 Fig. The negative USAF (R1DS1N, Thorlabs) test-chart was illuminated incoherently (M470L3 Thorlabs) and projected behind a glass sphere (Borosilicate material, n = 1.48, 110μm diameter, Cospheric LLC, USA). The images were captured using an Andor Zyla 5.5 sCMOS camera, while focusing through the sphere (see S7 Fig). Depicted are images from the experiment (Real) and the simulation. The difference images are calculated wrt. to the undistorted test-chart image, showing that the real sphere-induced image distortions are qualitatively reproduced by the simulation. (PDF) [file pcbi.1006079.s016.pdf]

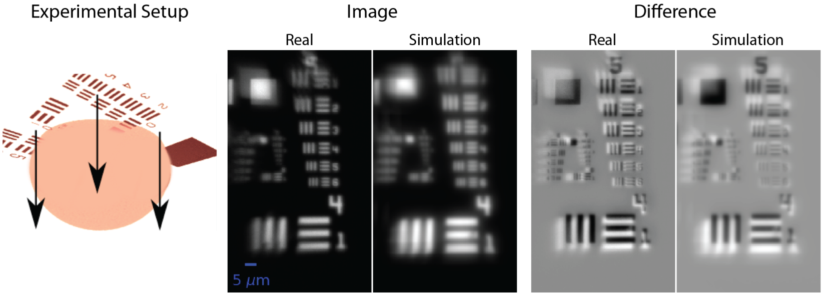

Supplementary Figure 8: Experimental micro-projection of a test-chart through a glass sphere and comparison with simulation. Experimental setup as in S7 Fig. The negative USAF (R1DS1N, Thorlabs) test-chart was illuminated incoherently (M470L3 Thorlabs) and projected behind a glass sphere (Borosilicate material, $n = 1.48$, $110\mu m$ diameter, Cospheric LLC, USA). The images were captured using an Andor Zyla 5.5 sCMOS camera, while focusing through the sphere (see S7 Fig.). Depicted are images from the experiment (Real) and the simulation. The difference images are calculated w.r.t. to the undistorted test-chart image, showing that the real sphere-induced image distortions are qualitatively reproduced by the simulation.
