# Supplementary material for: Biobeam—Multiplexed wave-optical simulations of light-sheet microscopy
Source: PLoS Comput Biol. 2018 Apr 13;14(4):e1006079. doi: 10.1371/journal.pcbi.1006079 (PMC5898703; doi:10.1371/journal.pcbi.1006079)
Supplement: S9 Fig — a) Guide-star assisted diffraction-limited focusing in scattering tissue model. b) Lateral translation of this aberration compensated beam leads to gradual degradation. c) Quantification of focus degradation via correlation C(Δx) vs. distance (dotted line) at different penetration depth (in mean free path). Agreement with the correlation function of a scattered plane wave (solid line) is most pronounced at higher penetration depth. Deviation at low penetration depth (1 mfp) agree with experimental observations [10]. (PDF) [file pcbi.1006079.s017.pdf]

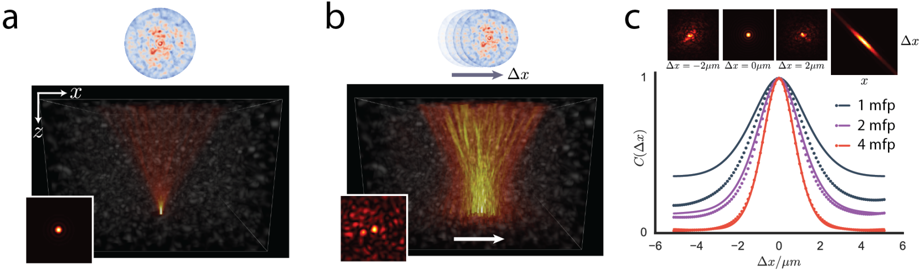

Supplementary Figure 9: Simulation of shift-shift memory effect. a) Guide-star assisted diffraction-limited focusing in scattering tissue model. b) Lateral translation of this aberration compensated beam leads to gradual degradation. c) Quantification of focus degradation via correlation $C(\Delta x)$ vs. distance (dotted line) at different penetration depth (in mean free path). Agreement with the correlation function of a scattered plane wave (solid line) is most pronounced at higher penetration depth. Deviation at low penetration depth (1 mfp) agree with experimental observations (Judkewitz, 2015).
