# Supplementary material for: Biobeam—Multiplexed wave-optical simulations of light-sheet microscopy
Source: PLoS Comput Biol. 2018 Apr 13;14(4):e1006079. doi: 10.1371/journal.pcbi.1006079 (PMC5898703; doi:10.1371/journal.pcbi.1006079)
Supplement: S10 Fig — a) A rectilinear ground truth stripe pattern occurs distorted when imaged through a sphere. b) Fitting a radial distortion map to simulation results of this scenario, allows one to fix the aberrations at the region of interest (green) as seen through the spherical cell phantom. (PDF) [file pcbi.1006079.s018.pdf]

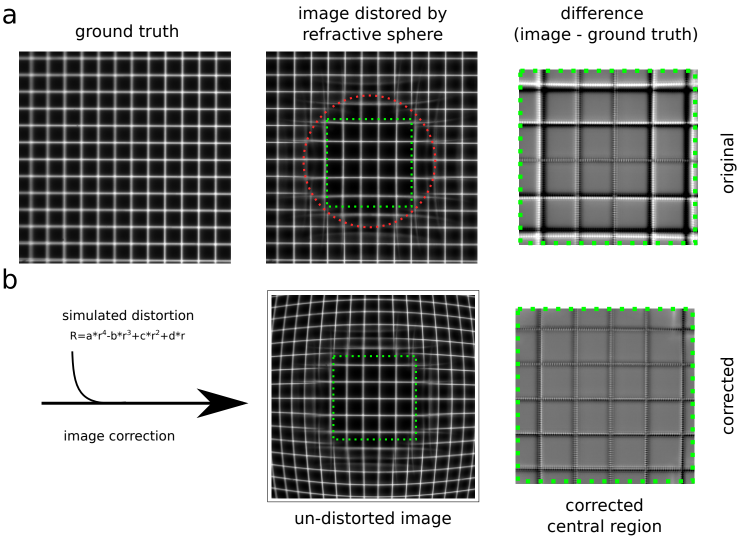

Supplementary Figure 10: a) A rectilinear stripe pattern occurs distorted when imaged through a sphere. b) Fitting a radial distortion map to simulation results of this scenario, allows one to fix the aberrations at the region of interest (green) as seen through the spherical cell phantom.
