# Supplementary material for: Biobeam—Multiplexed wave-optical simulations of light-sheet microscopy
Source: PLoS Comput Biol. 2018 Apr 13;14(4):e1006079. doi: 10.1371/journal.pcbi.1006079 (PMC5898703; doi:10.1371/journal.pcbi.1006079)
Supplement: S1 Table — (PDF) [file pcbi.1006079.s019.pdf]

Dimension ($x \times y \times z$)	Mie code (Gmmfield)	FDTD (Meep)	*biobeam* BPM
(128,128,128)	1314s	80 s	34 ms
(256,256,256)	10480s	790 s	81 ms
(512,512,512)	–	4800 s	154 ms
(1024,1024,1024)	–	–	440 ms

Supplementary Table 1: Runtimes of plane wave propagation through a given refractive index distribution of given dimensions.
